# Potential Use of Hazelnut Beverage in Water Kefir Fermentation: Effects of Sugar Addition on Physicochemical, Microbiological, Rheological, Bioactive, Volatile, and Allergenic Properties

**DOI:** 10.1111/1750-3841.71432

**Published:** 2026-08-31

**Authors:** Latife Betul Gul, Abdullah Akgun, Osman Gul

**Affiliations:** ^1^ Department of Food Engineering, Faculty of Engineering Ondokuz Mayis University Samsun Türkiye; ^2^ Department of Food Engineering, Faculty of Engineering Trakya University Edirne Türkiye; ^3^ Department of Food Engineering, Faculty of Engineering and Architecture Kastamonu University Kastamonu Türkiye

**Keywords:** allergenicity, fermentation, functional beverage, hazelnut cake, sustainability, water kefir

## Abstract

This study investigated the potential of hazelnut beverage, obtained from hazelnut cake through high‐pressure homogenization (100 MPa), for use in water kefir production. The effects of different sugar concentrations (2.5%, 5%, and 7.5%) on product properties and hazelnut allergenicity were also examined. Sugar addition significantly stimulated microbial growth, resulting in significant increases in *Lactobacilli*, *Lactococci*, acetic acid bacteria, and yeast counts compared to the control sample. At the end of fermentation, the pH values of all samples (4.32–4.54) were found to be below the food safety critical limit of pH 4.6. The best water‐holding capacity and physical stability were observed in the sample containing 5% sugar. Rheological analyses showed that the samples exhibited pseudoplastic behavior and that sugar addition increased viscosity. The addition of sugar contributed to an increase in the total phenolic content (11.26–15.15 mg GAE/100 mL) and antioxidant activity (DPPH: 16.77%–22.69%; ABTS: 42.68%–46.79%) at the end of fermentation. In the volatile aroma profile evaluation, 47 volatile compounds were tentatively identified based on mass spectral library matching, and increasing sugar concentration resulted in higher relative abundances of ethanol (6.72%–15.51%) and acetic acid (6.29%–13.88%). Allergen levels decreased from 16.6 µg/mL to 8.42–12.97 µg/mL with the addition of sugar. In conclusion, hazelnut cake, a byproduct with low added value, has been transformed into a new, functional, plant‐based, low allergenic, and consumer‐approved functional beverage. The developed hazelnut‐based water kefir offers a significant alternative for individuals with milk protein allergy, lactose intolerance, and vegan/vegetarian consumers.

**Practical Applications:**

Hazelnut cake is a low‐value by‐product from cold‐pressed hazelnut oil production. It can be transformed into a better functional beverage using water kefir fermentation. The hazelnut‐based water kefir offers a sustainable and eco‐friendly way to make use of food industry by‐products. It also meets the growing demand for plant‐based fermented drinks. Fermentation enhances the beverage's bioactive properties and lowers hazelnut allergen levels, increasing its potential for creating products with better functionality. In addition, the product serves as a great option for vegan and vegetarian consumers, as well as for those with lactose intolerance or milk protein allergies. The findings of this study may aid in creating new non‐dairy fermented drinks and help diversify sustainable food products in the beverage industry. Using 5% sugar supplementation could be particularly beneficial for industrial uses because of its positive effects on physical stability, water‐holding capacity, and overall product quality.

## 1 Introduction

The microbial and enzymatic transformations that occur during fermentation alter the nutritional value and sensory profile of raw materials, resulting in the emergence of desired product characteristics. Furthermore, fermentation increases the digestibility of foods, reduces undesirable compounds such as phytates and tannins, and enhances the bioavailability of plant‐based proteins by promoting proteolysis and converting proteins into amino acids (Alrosan et al. 2023). In recent years, the demand for the production and consumption of fermented foods and beverages has increased significantly due to their beneficial effects on health, nutritional properties, and sensory profiles (Dikmetas et al. 2026; Erem et al. 2026). Among these products, kefir is widely consumed as a functional fermented beverage and is recognized for its potential benefits to consumer health and well‐being (Cufaoglu and Erdinc 2023a).

Water kefir is a natural fermentation product formed by the symbiotic relationship of lactic acid bacteria (LAB), acetic acid bacteria (AAB), and yeasts (Wang et al. 2025). Structurally, it resembles milk kefir grains but develops in plant‐based sugar solutions rather than in milk, such as sugar water supplemented with dried fruits (e.g., figs or raisins), fruit juices, and other plant‐derived substrates used as fermentation media (Gokirmakli and Guzel‐Seydim 2022; Laureys and De Vuyst 2014). The microorganisms used in water kefir production primarily include *Lactobacillus*, *Leuconostoc*, and *Streptococcus* species, as well as yeasts such as *Saccharomyces* and *Hanseniaspora*. The diversity of this microflora varies depending on the fermentation conditions and the type of substrate used (Gulitz et al. 2011). During fermentation, the symbiotic relationships of these microorganisms produce lactic acid, CO_2_, ethanol, acetic acid, and various volatile aromatic compounds, resulting in a sparkling beverage with an acidic, sweet, slightly alcoholic taste, and yeast aroma (Fels et al. 2018).

Rising health concerns (milk protein allergies, lactose intolerance, high fat/cholesterol) and the growing popularity of vegan/vegetarian diets have increased interest in water kefir (Alrosan et al. 2023). With probiotic properties similar to milk kefir, it is a suitable alternative for vegans and individuals with lactose intolerance or milk allergies (Cufaoglu and Erdinc 2023b; Fiorda et al. 2017). In recent years, water kefir fermentation has been investigated using different substrates, including plant‐based solutions, fruit juices, and herbal infusions, to improve product characteristics such as microbial growth, aroma profile, and functional properties. (Fiorda et al. 2017). The literature reports the successful use of fruits such as apples, quinces, kiwis, etc., as well as vegetables such as carrots, melons, etc., in water kefir production (Dikmetas et al. 2026; Gökırmaklı et al. 2025; Gulhan et al. 2025; Nguyen et al. 2025; Papadopoulou et al. 2025). The use of plant‐based sources not only enriches the nutritional value of the fermented beverage but also affects microbial metabolism, polyphenol bioavailability, and the final product's sensory properties. However, studies on the use of nuts in water kefir production are quite limited. In this context, hazelnuts, especially hazelnut cake obtained after oil extraction, offer significant potential for the development of high‐value‐added products. Hazelnuts are rich in macronutrients such as carbohydrates (15%–18%), protein (11%–16%), and fat (58%–64%), as well as being an important source of vitamins, minerals, organic acids, phenolic compounds, and fat‐soluble bioactive substances (Alasalvar et al. 2009; Tas et al. 2019). Hazelnut cake, a cold‐press byproduct, is a nutritious component with high protein (43.77%), carbohydrate (25.20%), and fat (17.38%) content that can be evaluated in product development for human consumption (Gul et al. 2017). In recent years, studies on transforming hazelnut cake into high‐value‐added products have increased, and its potential for use as a milk substitute in beverage production and fermented products has been revealed (Atalar 2019; Gul et al. 2022).

With increased nut consumption, nut allergy has become one of the most common food allergies (Yin et al. 2024). Food allergies can lead to serious health problems such as urticaria, rhinitis, and anaphylactic shock, and avoiding the allergen is the only option when specific immunotherapy is not available (Acker et al. 2017; Fu et al. 2020). However, since completely avoiding allergens is not practically possible, reducing allergenicity through food processing methods is becoming increasingly important. Fermentation, unlike physical and chemical methods, is a biological process that can reduce allergenicity without negatively affecting the nutritional value and sensory properties of the food (L. Chen et al. 2012; El‐Ghaish et al. 2011). The reduction of allergenicity by microorganisms during fermentation occurs primarily through enzymatic hydrolysis and changes in allergenic epitopes. Microorganisms also possess immunomodulatory effects influencing antigen‐presenting cells, T cell responses, and intestinal barrier function. These processes may help reduce allergic reactions (Xu et al. 2024). Studies on peanut and cashew‐based beverages have demonstrated that fermentation can significantly reduce allergenicity and enhance protein digestibility (J. M. Chen et al. 2020; Mattison et al. 2020; Pi et al. 2020), while similar reductions have also been reported in other plant‐based fermented systems (Erem et al. 2026; Tang et al. 2023).

In this study, the potential of using hazelnut beverage, obtained from hazelnut cake through high‐pressure homogenization, in water kefir production was investigated. The physicochemical, microbiological, bioactive, and rheological properties, as well as the volatile component profile of hazelnut‐based water kefir, were determined. In addition, the effect of water kefir fermentation on hazelnut allergenicity was also revealed. With this comprehensive approach, the aim was to transform hazelnut cake into a high‐value‐added product and to develop a new plant‐based, functional, and reduced‐allergenic fermented beverage.

## 2 Material and Methods

### 2.1 Materials

Dehulled and skinless hazelnuts (*Corylus colurna*) were sourced from Gürsoy Hazelnut Production Factory (Ordu, Türkiye). Water kefir grains were obtained from Danem Inc. (Isparta, Türkiye), and commercial food‐grade sucrose was purchased from a local market in Samsun, Türkiye. Unless otherwise specified, all chemicals, reagents, and microbiological media were of analytical grade and purchased from Merck (Darmstadt, Germany) or Sigma‐Aldrich (St. Louis, MO, USA).

### 2.2 Production of Hazelnut Beverage

The production of hazelnut beverage from hazelnut cake was carried out according to the method proposed by Gul, Atalar, Mortas, et al. (2018a). Hazelnut cake (94.88% total solids, 43.92% protein, 34.81% carbohydrate, 11.09% lipid, and 5.06% ash) obtained after the removal of the shells and cold‐press oil extraction was used as the raw material for beverage production. First, hazelnut cake was ground into a fine powder using a Waring blender for 3 min. The powder was then dispersed in purified water to achieve a hazelnut‐to‐water ratio of 10% (w/v), previously identified as the optimal formulation in our earlier study (Gul et al. 2018), and homogenized using an Ultra‐Turrax homogenizer (IKA T25 Digital, Darmstadt, Germany) at 10,000 rpm for 10 min. To reduce the particle size of the resulting hazelnut beverage, increase the amount of water‐soluble dry matter, and obtain a sensorially acceptable product, a homogenization process was performed in a high‐pressure homogenizer (Pandaplus 2000, GEA Niro Soavi, Parma, Italy) at 100 MPa. The proximate composition of hazelnut beverage was determined as pH 6.48, 9.51% total solids, 4.29% protein, 0.96% lipid, and 0.53% ash.

### 2.3 Production of Hazelnut‐Based Water Kefir

In the production of hazelnut‐based water kefir, hazelnut beverage was used as the fermentation medium. The water kefir grains used for fermentation were obtained from Danem Inc. (Isparta, Türkiye), and contained *Acetobacter* spp., *Bifidobacterium* spp., *Gluconobacter* spp., *Lactobacillus* spp., *Lactococcus* spp., and *Streptococcus* spp. (Gökırmaklı et al. 2023). Fermentation was carried out in two stages. In the first stage, water kefir grains were inoculated into 5% (5 g/100 mL) sugar water, activated by incubating at 25°C for 24 h, and then the grains were separated by filtering into a sterile container. The activated grains were then transferred to amber‐colored glass bottles with caps (Bueno et al. 2021). In the second stage, fermentation was performed by adding 5% activated kefir grains to plain hazelnut beverage (control) and to hazelnut beverages supplemented with commercial food‐grade sucrose at concentrations of 2.5%, 5%, and 7.5%, which were pasteurized at 65°C for 30 min. The samples were left to ferment at 25°C for 72 h and after fermentation, the samples were stored in the refrigerator overnight before analysis (Gökırmaklı et al. 2023; Tireki 2025).

### 2.4 Determination of Proximate Compositions

The total solid content of the samples were determined using the gravimetric method, the protein content using the Kjeldahl method, the fat content using the Soxhlet method, and the ash content by burning at 550°C, based on the methods recommended by AOAC (2000). The pH of the hazelnut beverage and hazelnut‐based water kefir was determined using a calibrated pH meter (Eutech Cyberscan 2700, Ayer Rajah Crescent, Singapore). The color characteristics of kefir samples were measured using L* (lightness/whiteness), a* (red‐green axis), and b* (yellow‐blue axis) values, and a Minolta Chroma Meter (CR‐400, Osaka, Japan) colorimeter was used for these measurements

### 2.5 Microbiological Analysis

Microbiological analysis was performed using 0.85% physiological saline for serial dilutions. Total bacteria, LAB (lactobacilli and *Lactococci*), AAB, and yeasts were enumerated using spread or pour plate methods. Total bacteria were counted on plate count agar (PCA) (Merck Germany) medium at 30°C for 48 h, AAB on dextrose sorbitol mannitol (DSM) agar at 37°C for 24 h, lactobacilli on De Man, Rogosa, and Sharpe (MRS) agar under anaerobic conditions at 37°C for 48 h, *Lactococci* on M17 agar at 37°C for 48 h, and yeasts on potato dextrose agar (PDA) at 25°C for 48 h under aerobic conditions (Alrosan et al. 2023). Results were expressed as log_10_ cfu/mL based on the average at least three plates.

### 2.6 Water‐Holding Capacity, Physical Stability, and Firmness

The water‐holding capacities of kefir samples were determined according to the method reported by (Gul et al. 2023). For this purpose, approximately 10 g of homogenized kefir sample was weighed, transferred to a test tube, and centrifuged at 3250 × *g* for 10 min at 4°C. After centrifugation, the separated supernatant was weighed, and its water‐holding capacity (WHC) was calculated using Equation (1).

(1) $$\mathrm{WHC}\;\left(\%\right)=\left(1\;-\;\frac{W_{1}}{W_{2}}\right)\;\times \;100$$

The physical stability of the samples was assessed by measuring the amount of serum separated over 10 days of refrigerated storage. For stability measurement, graduated 15 mL centrifuge tubes were filled with the sample up to the line, and phase separation was determined during storage. Results are given as %.

Firmness was determined by a penetration test using a TA.XT Plus Texture Analyzer (Stable Micro Systems, Godalming, UK) equipped with a 35 mm cylindrical probe. Prior to analysis, samples (100 mL) were equilibrated at 20°C, and the probe penetrated the sample to a depth of 10 mm at a constant speed of 2 mm/s. Firmness was expressed as the maximum force (*g*) recorded during penetration (Gul et al. 2021).

### 2.7 Rheological Analysis

The rheological properties of hazelnut‐based water kefirs were determined using a Haake Mars III rheometer (Thermo Scientific, Germany). Measurements were performed using a conical plate geometry with a 2° conical angle, a 35 mm diameter, and a 0.105 mm measuring gap. A Peltier system was used for temperature control during the experiments, and all measurements were taken at 4°C. The samples were allowed to equilibrate at 4°C for 2 min before measurement (Gul et al. 2021). Flow characterization was performed using the flow ramp method, which is based on recording shear stresses by linearly increasing shear rates from 1 to 100 1/s. The obtained shear stress‐shear rate data were analyzed using the Ostwald‐de‐Waele (power law) model (Equation 2) to determine the viscosity behavior of the samples.

(2) $$\eta_{app}=K\gamma^{\left(n-1\right)}$$

where, *η*_app_ is the apparent viscosity (Pa.s), *γ̇* is the shear rate (1/s), *K* is the consistency index (Pa.sn), and *n* is the flow property index.

### 2.8 Determination of Total Phenolic Content and Antioxidant Activity

The total phenolic content of hazelnut‐based water kefir was determined using the Folin–Ciocalteu method, while antioxidant activity was evaluated by DPPH and CUPRAC assays. Prior to these analyses, bioactive compounds were extracted from the samples according to the method described by Sözeri Atik et al. (2021). For this, kefir samples (10 g) were extracted with 75% methanol and homogenized using an Ultra‐Turrax homogenizer. The mixtures were centrifuged at 8000 × *g* for 30 min at 4°C (Nuve, NF 800R, Turkey), and the supernatants were collected for analysis. For total phenolic content determination, 1 mL of extract was mixed with 2.5 mL of 0.2 N Folin–Ciocalteu phenol reagent and 2 mL of sodium carbonate, incubated in the dark for 30 min, and the absorbance was measured at 760 nm using a UV/VIS spectrophotometer (Shimadzu UV‐1800, Japan). Results were calculated using a Gallic acid standard curve and expressed as mg gallic acid equivalents (GAE)/100 mL sample (Sözeri Atik et al. 2021).

To determine the antioxidant activity by the DPPH method, 0.1 mL of extract was mixed with 4.9 mL of ethanolic DPPH solution. After incubation in the dark at room temperature for 30 min, absorbance was measured at 517 nm using a spectrophotometer. Antioxidant activity was expressed as percentage inhibition using the Equation (3).

(3) $$\mathrm{Activity}\;\left(\%\right)=\left[100\;-\;\left(\frac{\mathrm{Absorbance}\;\mathrm{of}\;\mathrm{sample}}{\mathrm{Absorbance}\;\mathrm{of}\;\mathrm{blank}}\right)\;\times \;100\right]\;$$

The ABTS radical‐scavenging capacity of the samples was determined following Azi et al. (2020). ABTS^+^ radicals were generated by reacting 7 mM ABTS with 2.45 mM K_2_S_2_O_8_ in the dark at room temperature for 16 h. The solution was diluted with ethanol, to an absorbance of 0.7 ± 0.02 at 734 nm, and equilibrated at 30°C. For the assay, 1.2 mL of ABTS^+^ solution was mixed with 0.3 mL of sample, reacted for 6 min, and absorbance was measured at 734 nm. Radical scavenging capacity was calculated using the following formula:

(4) $$\mathrm{Activity}\;\left(\%\right)=\left[\frac{\left(A_{c}\;-\;A_{S}\right)}{A_{C}}\right]\;\times \;100$$

where, *A_C_* and *A_S_* are the absorbance of the blank and samples, respectively

### 2.9 Determination of Aroma Profile

Volatile aroma components of hazelnut‐based water kefirs were determined using headspace analysis and Solid‐Phase Microextraction (SPME) technology and analyzed by gas chromatography–mass spectroscopy (GC–MS; Shimadzu GC‐2010, Scientific Instruments, Inc., Tokyo, Japan) equipped with a capillary column, 60 m, 0.32 mm i.d., 0.25 µm film thickness (Restek Stabilewax, Polyethylene glycol, USA) (Saygili et al. 2023). The mass spectrometer was operated in electron ionization (EI) mode at 70 eV. The ion source temperature and transfer line temperature were maintained at 200°C and 250°C, respectively, and mass spectra were acquired over a scan range of *m/z* 35–450. Samples (2 mL) were transferred to 15 mL glass vials for analysis, and the vials were placed in a water bath at 55°C. SPME fiber (2 cm, 50/30 µm DVB/Carboxen/PDMS, StableFlex, Supelco, Bellefonte, PA, USA) was used during headspace extraction. The fiber was left in the headspace environment for 60 min to adsorb volatile compounds. After extraction, the fiber was placed in the injection port of the gas chromatograph in split‐free mode, and volatile compounds were thermally desorbed at 250°C for 10 min. The temperature program for the GC–MS instrument was implemented as follows: initial temperature at 40°C, held for 1 min, then increased to 100°C at a rate of 7°C per minute and held for 5 min; subsequently, increased to 180°C at a rate of 2°C per minute and held for 1 min; finally, increased to 250°C at a rate of 15°C per minute and held for 4 min. Helium was used as the carrier gas, with a flow rate of 3 mL/min. Volatile compounds were tentatively identified by comparing their mass spectra with those contained in the NIST, Wiley, and FFNSC libraries. Because authentic reference standards and linear retention index (LRI) verification were not employed, the compound assignments should be regarded as tentative rather than definitive identifications. The objective of the volatile analysis was to compare relative differences among fermentation treatments rather than to provide definitive structural confirmation of individual volatile compounds. Quantification of volatile compounds was performed using a semi‐quantitative approach based on GC–MS peak area normalization, and results were expressed as relative peak area percentages (%). No internal standard was used. Therefore, the results represent relative abundances rather than absolute concentrations.

### 2.10 Determination of Allergenicity

For allergen analysis of hazelnut beverage and hazelnut‐based kefir samples, a commercial ELISA kit (SENSISpec ELISA Hazelnut, 96‐well) was used. This method allows the determination of the immunoreactivity of hazelnut protein residues in samples using a non‐competitive ELISA format. Before analysis, 1 mL of each sample was mixed with the salt‐Tris/HCl extraction buffer (0.05 M Tris, 0.2 M NaCl, 1 M HCl, pH 8.25) at a 1:5 ratios. The mixture was shaken for 2 min at room temperature, then centrifuged at 3800 rpm for 10 min at 15°C to obtain the supernatant. Prior to the immunoassay, soluble protein content in the supernatants was determined using the Bradford method with bovine serum albumin (BSA) as the standard, and the protein concentrations were adjusted to 25 ppm to ensure compatibility with the ELISA system. The ELISA assay was performed using microplates coated with anti‐hazelnut antibodies. The calibration curve was constructed using the standards provided with the commercial kit (10 and 100 ppm hazelnut protein) (kit reference standards), along with additional serial dilutions (30, 15, 5, and 2 ppm) prepared from the same reference material. A total of 100 µL of standards and sample extracts were added to the wells and incubated at room temperature for 20 min. After incubation, wells were washed three times with the kit‐provided washing solution. Subsequently, 50 µL of anti‐hazelnut biotin was added to each well and incubated for 20 min at room temperature, followed by washing. Detection was achieved using an avidin‐peroxide conjugate incubated at 25°C for 15 min. Excess reactants were removed by washing, after which bound peroxidase activity was determined by the development of a blue color over 45 min upon addition of 3,3′,5,5′‐tetramethylbenzidine (TMB) substrate. The reaction was terminated with a stop solution until the color changed from blue to yellow, and the absorbance value was measured at 450 nm using a BenchMark Plus microplate reader (Bio‐Rad) controlled by Microplate Manager software.

### 2.11 Statistical Analyses

Three independent fermentation batches were produced under identical conditions. Unless otherwise stated, all analytical measurements were performed in duplicate, and the reported values represent the mean ± standard deviation. Statistical analysis of all obtained data was performed using SPSS Statistics 21.0 (SPSS Inc., Chicago, Illinois, USA) program, and one‐way analysis of variance (ANOVA) was applied for each parameter. Significant differences between means were determined using Duncan's multiple comparison test. Rheological analyses were evaluated using the Rheowin 4 Data Manager software package (version 4.20, Haake).

## 3 Result and Discussions

### 3.1 Microbiological Properties

**FIGURE 1:**
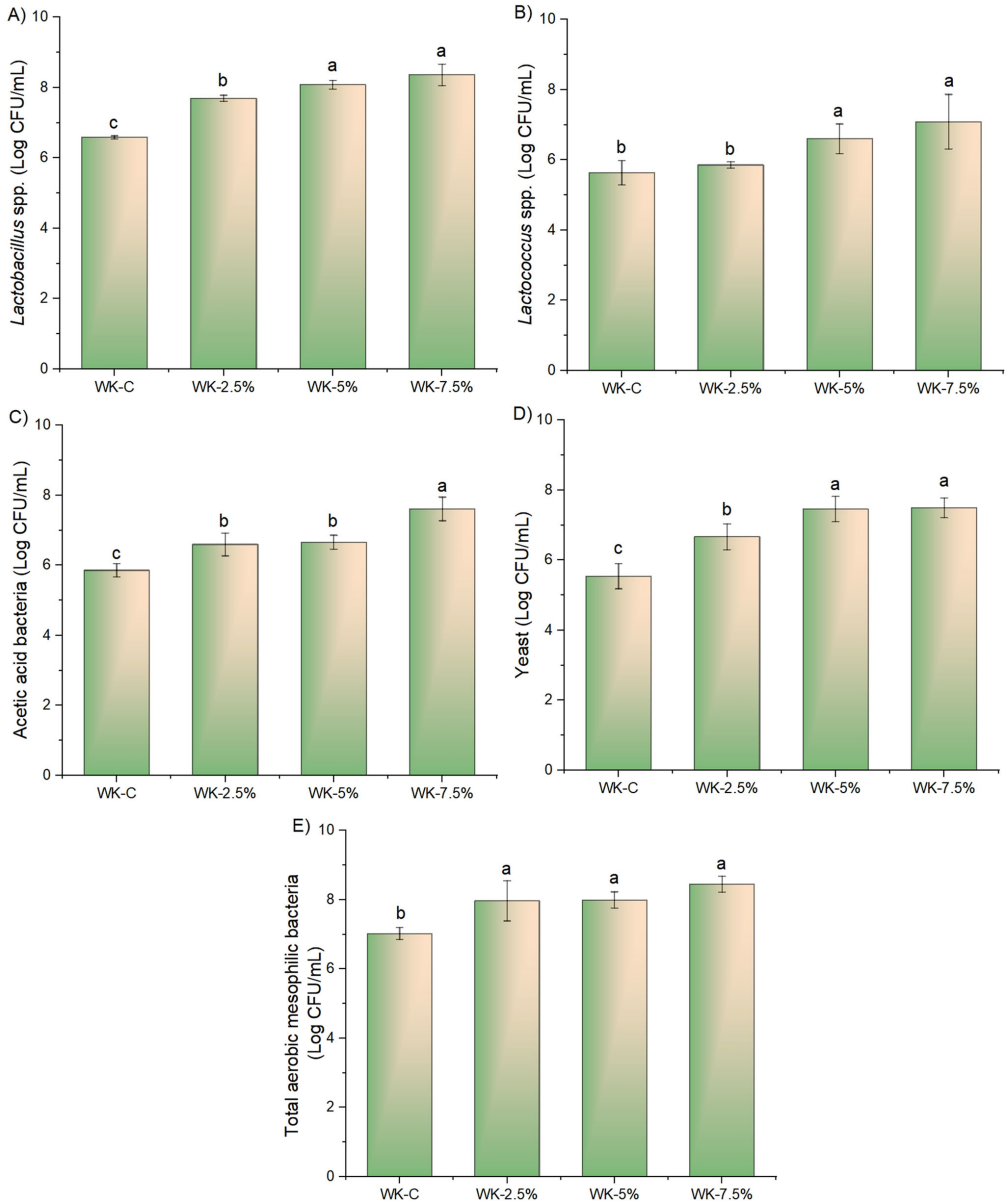
Microbiological contents of hazelnut‐based water kefir samples (Log CFU/mL) (WK‐C: sugar‐free hazelnut‐based water kefir [Control]; WK‐2.5%: hazelnut drink‐based water kefir with 2.5% sugar; WK‐5%: hazelnut drink‐based water kefir with 5% sugar; WK‐7.5%: hazelnut drink‐based water kefir with 7.5% sugar).

The microbial diversity of water kefir consists mainly of a stable consortium composed of LAB, AAB, and yeasts. Different species have been found to exist in symbiotic relationships, surviving or multiplying by sharing their bioproducts as an energy source or growth‐stimulating factor (Fiorda et al. 2017). *Lactobacillus*, *Lactococci*, AAB, yeasts, and total bacterial counts for hazelnut‐based water kefir samples are shown in Figure 1. At the end of fermentation, lactobacilli count ranged from 6.58 to 8.36 log cfu/mL (*p* < 0.05), with the lowest in the control and highest in the 7.5% sugar‐containing samples. Sugar addition significantly increased *Lactobacillus* counts (*p* < 0.05; Figure 1a). Literature reports lactobacilli count in water kefir range between 5 and 9.5 log cfu/mL, depending on the nutrient source (soy, whey, various fruit and vegetable juices, sugar water, etc.). Although sucrose alone is generally insufficient to support lactobacilli growth (Koh et al. 2018), in mixed water kefir cultures, it is first hydrolyzed into glucose and fructose, which are more readily utilized for growth (Laureys and De Vuyst 2014). In this study, the combination of hazelnut‐derived nutrients with sugar created a favorable environment for lactobacilli proliferation.

In the control kefir sample, the *Lactococcus* levels were 5.63 log cfu/mL, while in sugar‐supplemented samples, they ranged from 5.85 to 7.09 log cfu/mL (Figure 1b). No significant difference was observed between the control and the 2.5% sugar sample (*p* > 0.05), whereas samples with medium and high sugar levels showed significantly higher *Lactococci* counts (*p* < 0.05). These results regarding are consistent with previous studies, which indicate that sugar availability enhances the metabolic activity of *Lactococcus* spp. in water kefir systems (Corona et al. 2016; Gökırmaklı et al. 2023).

The AAB counts ranged from 5.95 to 7.81 log cfu/mL (Figure 1c). Compared with the control sample, AAB numbers were higher in samples with added sugar, and the addition of sugar significantly increased AAB counts (*p* < 0.05). This aligns with findings by Limbad et al. (2023), who reported higher AAB counts in sugar‐supplemented water kefir. AAB growth is supported by LAB‐derived lactic acid and yeast‐derived metabolites, which create favorable redox and substrate conditions for acetic acid production (Martínez‐Torres et al. 2017).

Yeast counts were 5.54 log cfu/mL in the control samples, increasing to 6.49, 6.66, and 7.81 log cfu/mL in kefirs with low‐, medium‐, and high‐sugar addition, respectively (Figure 1d). Thus, higher sugar levels led to increased yeast proliferation. Similar trends have been reported by Limbad et al. (2023) and Laureys and De Vuyst (2014), with yeast counts in water kefir typically ranging from 3.3 to 8 log cfu/mL (Corona et al. 2016; Magalhães et al. 2011; Nascimento et al. 2022). Furthermore, as reported by Laureys and De Vuyst (2014), yeast counts were lower than LAB counts in our kefir samples.

In water kefir, LAB, AAB, and yeasts coexist symbiotically. In the presence of sucrose, yeasts hydrolyze sucrose into glucose and fructose using invertase activity, thereby providing fermentable sugars for LAB and AAB metabolism (Fiorda et al. 2017). However, during fermentation, an acidic environment favorable to yeast growth is created by the bacterial metabolic activity. Yeasts also provide vitamins and soluble nitrogen compounds essential for bacterial development (Magalhães et al. 2011). On the other hand, some studies have reported that phenolic and antimicrobial compounds in hazelnut beverages can inhibit microbial growth (Atalar 2019; Comak Gocer and Koptagel 2023). In the present study, sugar addition improved carbon availability and enhanced microbial metabolic balance, thereby mitigating potential inhibitory effects and supporting overall fermentation performance.

### 3.2 Physicochemical Properties

The physicochemical properties of control and hazelnut‐based water kefir with different sugar levels are summarized in Table 1. After fermentation, the total solids ranged from 8.86% to 14.27%, with the lowest value in the control and significantly higher values in sugar‐supplemented samples (*p* < 0.05). The reduction in total solids is consistent with the microbial consumption of fermentable substrates during fermentation, as previously reported for fermented beverages (Araújo et al. 2023). Similar trends have been reported in previous water kefir studies (Gökırmaklı et al. 2023; Rejdlova et al. 2023), as well as in rice, almond, and soy‐based beverages (Tu et al. 2019; Ustaoğlu‐Gençgönül et al. 2024). These changes are consistent with the hydrolysis of sucrose into glucose and fructose by yeast invertase activity (Dickinson and Kruckeberg 2006) and subsequent microbial metabolism of monosaccharides through LAB and yeast fermentation pathways (Laureys and De Vuyst 2014).

**TABLE 1:** Physicochemical properties of hazelnut‐based water kefir samples.

Properties	WK‐C	WK‐2.5%	WK‐5%	WK‐7.5%
Total solids (%)	8.86 ± 0.05^d^	11.04 ± 0.04^c^	12.69 ± 0.49^b^	14.27 ± 0.26^a^
Protein content (%)	4.31 ± 0.17^a^	4.27 ± 0.09^a^	4.24 ± 0.11^a^	5.22 ± 0.24^a^
Oil content (%)	0.89 ± 0.06^a^	0.91 ± 0.04^a^	0.88 ± 0.07^a^	0.92 ± 0.05^a^
Ash content (%)	0.46 ± 0.03^a^	0.44 ± 0.02^a^	0.45 ± 0.08^a^	0.47 ± 0.05^a^
pH	4,54 ± 0,04^a^	4,47 ± 0,03^b^	4,43 ± 0,02^b^	4,31 ± 0,06^c^
L*	78.33 ± 0.05^a^	78.1 ± 0.08^a^	77.56 ± 0.05^b^	76.92 ± 0.1^c^
a*	−1.78 ± 0.01^a^	−1.75 ± 0.01^a^	−1.51 ± 0.01^b^	−1.32 ± 0.01^c^
b*	4.76 ± 0.02^a^	4.83 ± 0.06^a^	4.84 ± 0.05^a^	4.74 ± 0.06^a^

*Note*: Means shown in different lowercase letters on the same row indicate significant differences between samples (*p* < 0.05). WK‐C: sugar‐free hazelnut‐based water kefir (Control); WK‐2.5%: hazelnut drink‐based water kefir with 2.5% sugar; WK‐5%: hazelnut drink‐based water kefir with 5% sugar; WK‐7.5%: hazelnut drink‐based water kefir with 7.5% sugar.

Ash content ranged from 0.45% to 0.47%, with no significant differences between samples (*p* > 0.05), indicating that ash originated from hazelnut cake rather than added sugar. Similarly, fat content did not differ significantly between samples (*p* > 0.05).

The pH of the kefir samples ranged from 4.54 to 4.32 at the end of the fermentation period. The lowest pH was observed in the sample with 7.5% added sugar, while the highest pH was observed in the control sample. As expected, the acids produced by the metabolism of sucrose by the kefir grain microbiota during fermentation caused a decrease in pH. The final pH values of various plant‐based water kefirs ranged from 3.58 to 5.41 (Gökırmaklı et al. 2023; Pinto et al. 2020; Ustaoğlu‐Gençgönül et al. 2024), and our results are consistent with the these findings. The pH is an important parameter that provides general information about a food's sensory and structural properties and is also a critical indicator of food safety. It is stated that the pH of the product should be kept below 4.6 reducing the risk of *Clostridium botulinum* growth (Tanaka 1982). Most water kefirs are reported below pH 4.5, making them relatively safe against potential pathogenic microorganisms (Lynch et al. 2021). Overall, the hazelnut‐based beverage provides nutrients that support the growth of kefir microbiota and helps achieve the desired low pH, ensuring microbiological safety while maintaining sensory quality. Sugar addition further enhances fermentation, promoting microbial proliferation and facilitating the product's safe and consistent pH.

The L* values of the kefir samples were determined to be between 76.91 and 78.33, and the addition of sugar to the hazelnut beverage caused a decrease in the L* value of the samples and thus resulted in a partially darker colored kefir. In contrast, the a* values of the samples increased with sugar content, but no significant difference was observed in the b* value. All kefir samples showed negative a* values, indicating the presence of green tones (Paredes et al. 2022). It is not expected that the sugar used in hazelnut beverage production, by dissolving and becoming transparent, directly contributes significantly to these changes in L* and a* values. Therefore, the source of the changes is thought to be primarily related to biochemical and microbial processes occurring during fermentation rather than the direct effect of sugar itself. Considering the 72‐h fermentation period, it seems quite plausible that reducing sugars present in the environment or that may occur during this process, and free amino groups present in the hazelnut beverage, may interact, and therefore non‐enzymatic browning reactions such as the Maillard reaction may potentially occur under these conditions. However, it should be noted that the formation of Maillard reaction products was not directly measured in this study, and therefore this mechanism is proposed as a possible contributing factor rather than a confirmed pathway. The possibility of the Maillard reaction occurring in the production of quinoa‐containing water kefir has also been demonstrated by Palacios‐Castillo et al. (2025). Furthermore, the metabolic activities of yeasts and bacteria in water kefir grains not only affect the aroma and functional properties of the product (Spizzirri et al. 2023) but can also lead to changes in the color of the final product through the metabolites formed (Tireki 2025). Thus, microbial metabolism is considered a primary contributor to the observed color changes in the present study.

### 3.3 WHC, Physical Stability, and Firmness

**FIGURE 2:**
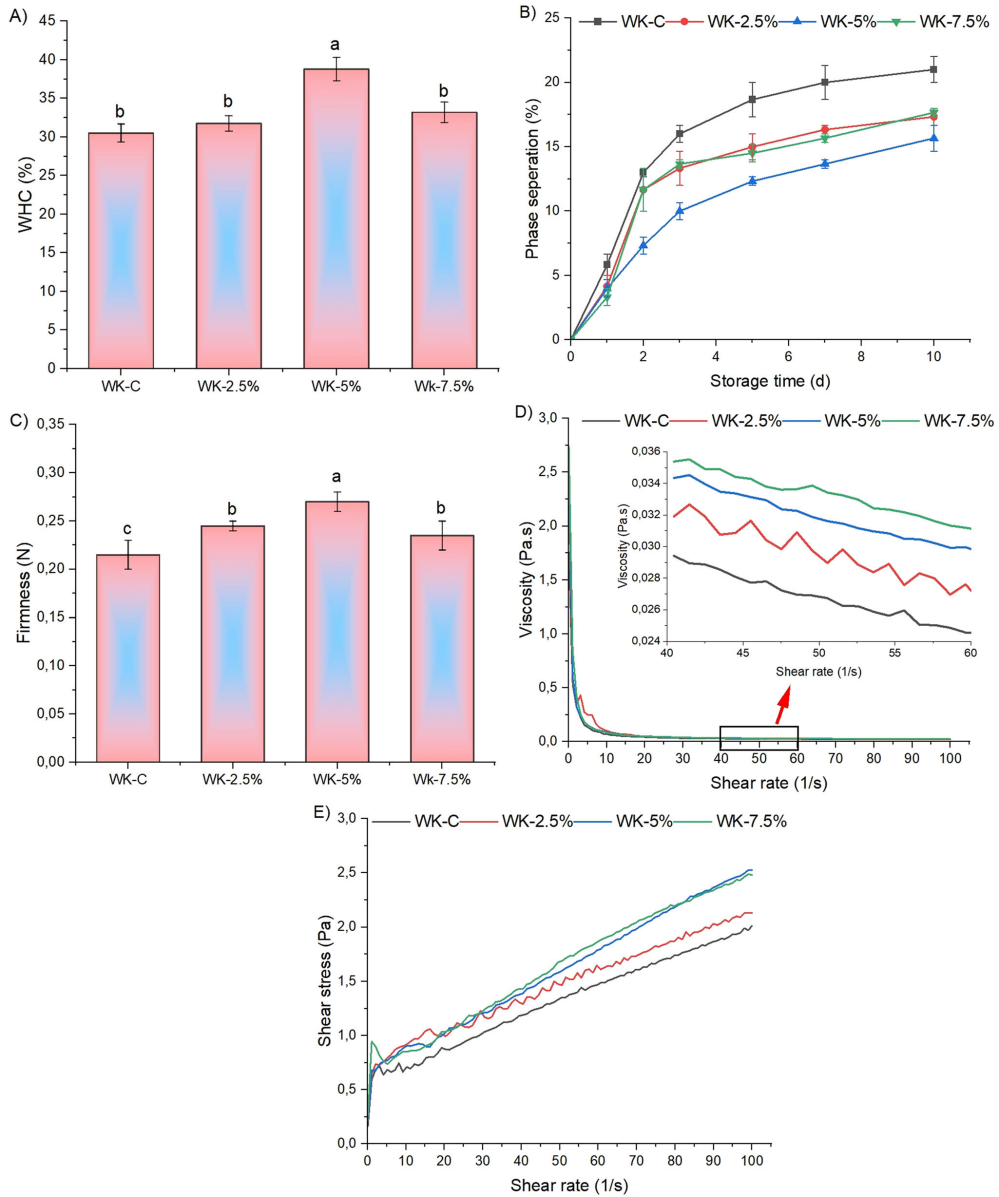
WHC (a), physical stability (b), firmness (c), and flow behavior of hazelnut‐based water kefir samples (WK‐C: sugar‐free hazelnut‐based water kefir [Control]; WK‐2.5%: hazelnut drink‐based water kefir with 2.5% sugar; WK‐5%: hazelnut drink‐based water kefir with 5% sugar; WK‐7.5%: hazelnut drink‐based water kefir with 7.5% sugar).

WHC and physical stability of hazelnut‐based water kefir were evaluated by centrifugation and measuring phase separation over 10 days of storage, respectively. WHC values ranged from 30.54% to 38.79% (Figure 2a), higher than those reported for almond (13.35%–22%) or soy based kefirs (15.30%–21.85%), respectively (Atik et al. 2020), likely due to the chemical composition of hazelnut beverage, fermentation conditions, and carbohydrate content. During fermentation, microbial activity metabolized carbohydrates, including soluble fibers such as starch and β‐glucans, into simpler sugars and organic acids (Bernat et al. 2015; Sadiq Butt et al. 2008), while plant proteins and insoluble fibers helped maintain gel structure and water retention (Gul et al. 2021; Patra et al. 2022).

The control sample had a WHC of 33.21%, which increased to 38.79% with 5% sugar addition, while higher sugar levels reduced WHC. These differences can be explained by the dual effect of sugar concentration on fermentation dynamics and matrix structure. At moderate levels (5%), sugar likely enhanced microbial activity and exopolysaccharide production, improving WHC, whereas at higher concentrations (7.5%), excessive osmotic pressure and altered acid production may have weakened the protein‐polysaccharide network, leading to reduced WHC. Generally, WHC is influenced by the microbial composition of the kefir culture, particularly the acid‐producing activity of the bacteria (Torre et al. 2003). In the present study, variations in sugar concentration during fermentation affected microbial counts and consequently the pH of the samples. The hazelnut‐based water kefir sample with the lowest WHC had a pH of approximately 4.3, which is further from the isoelectric point of hazelnut proteins (approximately pH 4.5) compared to other samples (Gul, Atalar, Saricaoglu, et al. 2018). Acidification of plant‐based systems can destabilize proteins and impair gel formation, thereby increasing serum separation (Uruc et al. 2022).

Physical stability during 10 days of storage (Figure 2b) showed that water separation increased in all samples over time. On Day 1, water separation in the control sample was 5.83%, while sugar‐containing samples ranged from 3.33% to 4.16%. By Day 3, the control reached 16%, whereas the 5% sugar sample remained at 10%. The 7.5% sugar sample initially had low separation but increased rapidly, second only to the control. After Day 3, separation in the sugar‐containing samples stabilized, while in the control, it slowed only after Day 5. At the end of storage, the highest phase separation was 21% in the control, and the lowest was 15.16% in the 5% sugar sample, with no significant differences among sugar‐added samples (*p* > 0.05). These differences among samples can be attributed to the balance between microbial exopolysaccharide production and acid‐induced protein destabilization, both of which are strongly influenced by sugar concentration.

Both the hazelnut beverage and sugar concentration significantly influenced firmness (*p* < 0.05). The control sample exhibited a gel strength of 0.215 N, while sugar addition increased this value by up to 28%, reaching 0.275 N. This increase is likely due to the higher total dry matter content resulting from sugar addition, consistent with Mohan et al. (2020), who reported a positive correlation between firmness and total solids. In addition, moderate sugar levels may have enhanced microbial exopolysaccharide formation, strengthening the gel network, whereas excessive sugar may have limited this effect by altering microbial metabolism and reducing structural cohesion. In contrast, some studies have shown that enriching fermented beverages with plant or agricultural by‐products can weaken gel networks and reduce gel strength. For instance, Öztürk et al. (2018) reported that adding hawthorn flour to yogurt reduced its firmness. In the present study, the observed increase in gel strength may also be attributed to the gel formed by hazelnut proteins interacting with water. Furthermore, increased sugar content may have promoted microbial activity, enhancing the production of exopolysaccharide‐like compounds and thereby strengthening the gel structure.

### 3.4 Rheological Properties

The flow behavior characteristics of control and hazelnut‐based water kefir samples with varying sugar levels were examined, and the results are presented in Figure 2d. The data indicate that the viscosity of all kefir samples decreases significantly with increasing shear rate, demonstrating shear‐thinning behavior. In other words, the system's resistance to flow diminishes as the shear rate increases. This type of behavior is characteristic of non‐Newtonian fluids and is frequently observed in complex, network‐forming food systems. It is well established that the non‐Newtonian rheological behavior of kefir and similar fermented beverages arises from the three‐dimensional structural network formed by polysaccharides, proteins, and microbial metabolites, particularly exopolysaccharides (EPS) produced by LAB. The shear‐thinning behavior of kefir systems has been widely reported in the literature, and similar findings have been confirmed in our previous studies (Gul et al. 2022, 2023). These observations indicate that the internal structure of kefir is highly sensitive to mechanical forces.

Examination of the shear stress–shear rate curves (Figure 2e) showed that shear stress increased with increasing shear rate in all samples. The non‐linear relationship between shear stress and shear rate indicates non‐Newtonian flow behavior and suggests the presence of structural interactions within the kefir matrix. This suggests that the kefir system possesses a gel‐like or weak gel‐like structure. Initially, greater force is needed to disrupt weak bonds and the three‐dimensional network. Once these bonds are broken and the structure partially disintegrates, the structural elements progressively aligned with the flow direction, resulting in lower apparent viscosity and easier flow. Furthermore, sugar supplementation affected the rheological response of the samples. The WK‐5% and WK‐7.5% samples exhibited higher shear stress values throughout most of the shear rate range compared with WK‐C and WK‐2.5%, indicating greater resistance to flow. The higher shear stress values observed in these samples are consistent with their elevated viscosity values and suggest the formation of a stronger internal network. Such behavior may be attributed to increased soluble solids and enhanced EPS production during fermentation. Similar rheological characteristics have been reported for other fermented and weak‐gel food systems (Mitra and Ghosh 2019). These findings reveal that the flow behavior of nut‐based water kefir exhibits typical non‐Newtonian characteristics resulting from complex structural interactions.

Measurements performed in the low shear rates (1–10 1/s) showed that the highest viscosity value was observed in the sample containing 7.5% sugar. In the same shear rate range, the viscosity values of the other samples were relatively similar, with only minor differences. This can be attributed to the reduced disruption of the internal structure at low shear rates, allowing dissolved solids, particularly in samples with higher sugar concentrations, to exert a more pronounced effect on viscosity. When the shear rate was held constant at 50 1/s, viscosity values ranged from 0.021 to 0.034 Pa·s (Table 2). Under these conditions, sugar‐supplemented samples exhibited a significant increase in viscosity compared to the control. This increase is likely due to the higher total dissolved solids content resulting from sugar addition. Indeed, Yanto (2015) noted that increased total dissolved solids are a primary factor contributing to higher viscosity in kefir samples. The presence of dissolved solids restricts the free movement of water, thereby increasing resistance to flow and raising viscosity. Similar findings were reported by Hastrawirid et al. (2024), who observed that increasing sucrose concentration in water kefir supplemented with *Hibiscus sabdariffa* L. raised the Brix value and, consequently, viscosity due to higher dissolved solids. Moreover, the increase in viscosity may also be related to the incomplete metabolism of sugars by microorganisms during fermentation. When high‐sugar concentrations are used, yeast and bacterial cultures may not fully utilize the available substrate, leading to the accumulation of unconverted sugars in the medium. This increases total dissolved solids and, in turn, viscosity. In addition, as noted by Abdul et al. (2018), LAB in water kefir convert only about 30% of available sugar into lactic acid, leaving the remaining 70% as residual sugar. This is particularly significant in high‐sugar environments, where substantial amounts of dissolved sugar remain post‐fermentation, affecting the density and total solid content, and thereby influencing the rheological properties of water kefir.

**TABLE 2:** Rheological properties of hazelnut‐based water kefir samples.

Properties	WK‐C	WK‐2.5%	WK‐5%	WK‐7.5%
*η*_app_ (Pa.s)	0.022 ± 0.001c	0.028 ± 0.001b	0.029 ± 0.001b	0.034 ± 0.002a
*K*	0.423 ± 0.025b	0.486 ± 0.027a	0.454 ± 0.045a	0.491 ± 0.046a
*N*	0.346 ± 0.022a	0.335 ± 0.014a	0.349 ± 0.006a	0.363 ± 0.01a
*R* ^2^	0.991 ± 0.001	0.996 ± 0.001	0.998 ± 0.001	0.991 ± 0.001

*Note*: Means shown in different lowercase letters on the same row indicate significant differences between samples (*p* < 0.05). WK‐C: sugar‐free hazelnut‐based water kefir (Control); WK‐2.5%: hazelnut drink‐based water kefir with 2.5% sugar; WK‐5%: hazelnut drink‐based water kefir with 5% sugar; WK‐7.5%: hazelnut drink‐based water kefir with 7.5% sugar. *η*_app_: apparent viscosity at 50 1/s shear rate; *K*: consistency index; *n*: flow behavior index (dimensionless); *R* ^2^: determination coefficient of Equation (2).

To explain the shear force–shear rate relationship, the Ostwald–de Waele (power‐law) model was applied to the rheological data. This model is widely used to describe the rheological properties of non‐Newtonian food systems and characterizes flow behavior through the consistency coefficient (*K*) and flow behavior index (*n*). The model demonstrated an excellent fit to the experimental data, with coefficients of determination (*R* ^2^) exceeding 0.99 for all samples, indicating that the power‐law model accurately describes the flow behavior of hazelnut‐based water kefir. Statistical analysis revealed that sugar concentration significantly affected both model parameters (*p* < 0.05). Variations in sugar levels influenced both the consistency coefficient (*K*) and the flow behavior index (*n*), with notable differences among samples. It is hypothesized that increased sugar content raises total dissolved solids, thereby enhancing flow resistance and increasing the consistency coefficient. All fermented beverage samples exhibited flow behavior index values (*n*) below 1, confirming non‐Newtonian, pseudoplastic (shear‐thinning) behavior. An *n* < 1 indicates that viscosity decreases and resistance to flow diminishes as shear rate increases. This pseudoplastic behavior is typically associated with structural changes within the system under shear, where the internal structure rearranges or breaks down in response to applied force.

### 3.5 Total Phenolic Content and Antioxidant Activity

**FIGURE 3:**
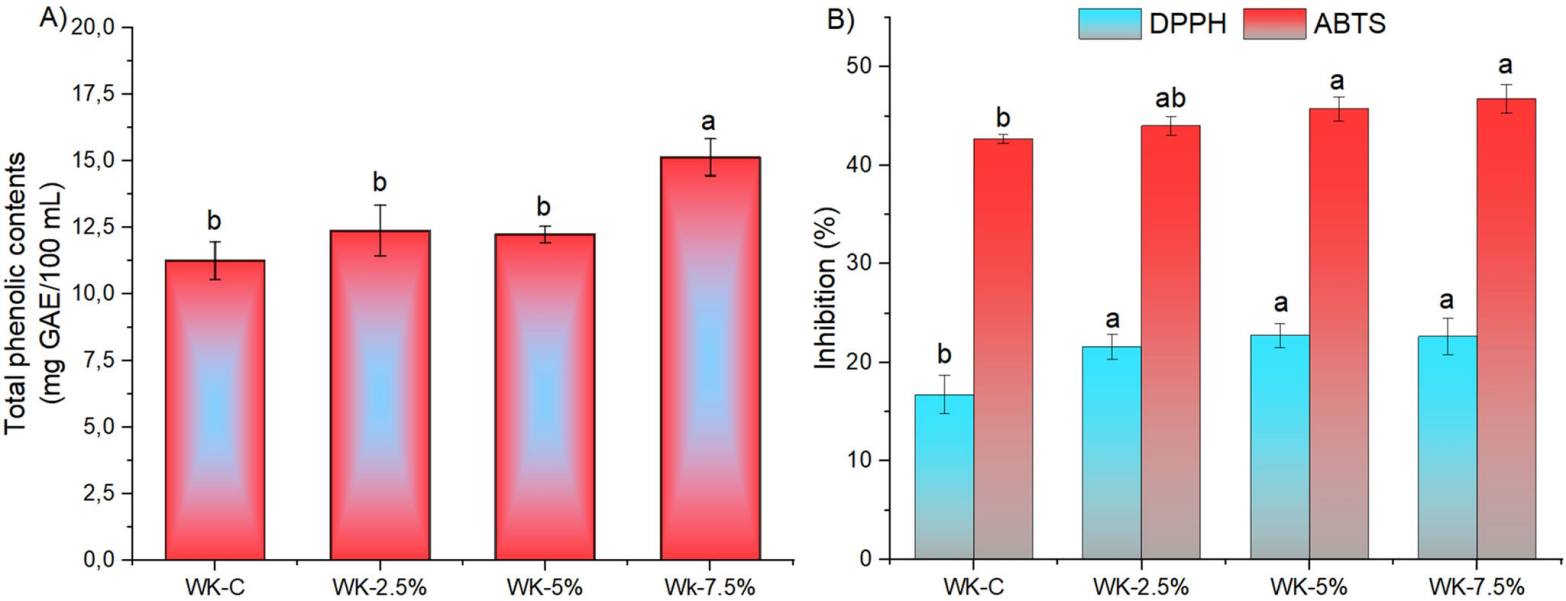
Total phenolic content (a) and antioxidant activity of hazelnut‐based water kefir samples (WK‐C: sugar‐free hazelnut‐based water kefir (Control); WK‐2.5%: hazelnut drink‐based water kefir with 2.5% sugar; WK‐5%: hazelnut drink‐based water kefir with 5% sugar; WK‐7.5%: hazelnut drink‐based water kefir with 7.5% sugar).

Phenolic compounds are widely recognized as key contributors to the bioactive properties of fermented beverages, and their content varies considerably depending on the source material. The total phenolic content of control and hazelnut‐based water kefir samples with varying sugar concentrations is presented in Figure 3a. As shown, total phenolic content ranged from 11.26 to 15.15 mg GAE/100 mL. Hazelnuts are a source of various polyphenols, including tocopherols (particularly α‐ and γ‐tocopherols), gallic acid, p‐hydroxybenzoic acid, epicatechin, sinapic acid, and quercetin, and are considered nutritionally valuable (Ozturkoglu‐Budak et al. 2016). Moreover, by‐products from the hazelnut oil industry, such as hazelnut cake, can serve as significant sources of phenolic compounds when used as a base for beverages (Atalar et al. 2021). However, the phenolic content of hazelnut cake and consequently hazelnut beverage may vary depending on the processing method. For instance, Tsai et al. (2018) reported that high‐pressure processing did not adversely affect the total phenolic and flavonoid levels of hazelnut beverage. In addition, thermal processing such as pasteurization in sugar‐supplemented systems may lead to the formation of Maillard reaction products, particularly melanoidins, which may react in Folin–Ciocalteu assays, thereby appearing as an increase in “apparent” total phenolic content rather than representing newly formed phenolic compounds (Kitchen and Williamson 2024). A similar increase in total phenolic content probably associated with Maillard reaction products has also been reported by Sagdic et al. (2012).

In hazelnut‐based water kefir production, increasing sugar concentration was associated with a slight increase in total phenolic content in the samples. However, no statistically significant difference was observed among samples except for the one containing 7.5% sugar (*p* > 0.05). In general, total phenolic content increased in parallel with sugar concentration, likely due to the combined effects of enhanced microbial metabolism and the contribution of Maillard‐derived antioxidant compounds formed during thermal processing. Furthermore, the literature indicates that the presence of symbiotic microorganisms during fermentation may influence phenolic compound content (La Torre et al. 2025; Teixeira Oliveira et al. 2023). According to La Torre et al. (2025), the observed increase in total phenolic content is likely attributable to the initial metabolic activity of mixed microorganisms, which hydrolyze polyphenols from bound to free forms within the first 24 h of fermentation. The higher phenolic levels in sugar‐supplemented samples could also be related to enhanced microbial activity and the presence of LAB exhibiting β‐glucosidase activity, which facilitates the hydrolysis of bound phenolic compounds into their free forms (Michlmayr and Kneifel 2014; Prado et al. 2015). Conversely, some studies report a decrease in phenolic content during fermentation, which has been attributed to the utilization of flavonoid‐containing phenolic compounds as nutrient sources by LAB (Esatbeyoglu et al. 2023). These contrasting findings suggest that variations in phenolic content may arise from differences in the microbiota composition of kefir grains or cultures, influenced by factors such as grain origin, fermentation conditions, substrate type, and culturing methods (Prado et al. 2015).

Oxidative stress results from an imbalance between prooxidants and antioxidant defenses, leading to cellular damage. In addition to endogenous antioxidant enzymes, dietary antioxidants can help neutralize free radicals (Pisoschi and Pop 2015). Therefore, the antioxidant capacity of hazelnut‐based water kefir was evaluated in this study in relation to its bioactive composition. In this context, the antioxidant properties of hazelnut‐based water kefir samples were evaluated using DPPH and ABTS radical scavenging assays, with results presented in Figure 3b. DPPH radical scavenging activity ranged from 16.77% to 22.69%, while ABTS radical scavenging activity ranged from 42.68% to 46.79%. Sugar‐supplemented kefir samples exhibited significantly higher DPPH and ABTS radical scavenging activities compared to the control (*p* < 0.05). However, no statistically significant differences were observed among the sugar‐containing samples themselves (*p* > 0.05). Consistent with trends in total phenolic content, the observed increase in antioxidant activity may be related to fermentation‐driven changes in bioactive compound availability. In addition, increased microbial growth at higher sugar levels may have contributed indirectly to antioxidant potential through metabolic activity of LAB, which can produce antioxidant enzymes, exopolysaccharides, and bioactive peptides (Amaretti et al. 2013; Mu et al. 2019; Wu et al. 2023). Some LAB have also been reported to exhibit reactive oxygen species scavenging capacity (Azi et al. 2020). Furthermore, fermentation‐related pH changes and enzymatic hydrolysis processes may also play a role in modifying antioxidant activity by altering the structure and availability of bioactive compounds (Safak et al. 2023).

### 3.6 Volatile Compounds

GC–MS analysis was conducted to identify differences in the volatile aroma compounds in control and hazelnut‐based water kefir samples with different sugar levels, and the results are presented in Table 3. A total of 48 volatile compounds were detected in the hazelnut‐based water kefir samples. These compounds, comprising 14 alcohols, 10 organic acids, 5 ketones, 7 aldehydes, 9 esters, and 3 compounds from other chemical classes, were tentatively identified by HS‐SPME‐GC‐MS using mass spectral library matching. Accordingly, the volatile profile should be interpreted as a comparative fingerprint of the samples rather than as definitive qualitative identification of individual aroma compounds. Overall, both the base ingredients of hazelnut‐based water kefir and sugar concentration significantly influenced the aroma profile. As shown in Table 3, one of the most notable findings is the increase in ethanol and acetic acid concentrations with rising sugar levels. In the control sample, ethanol and acetic acid were present at 6.72% and 6.29%, respectively, while in the high‐sugar sample, these values increased markedly to 15.51% and 13.88%. This trend suggests enhanced fermentative activity under higher sugar conditions. This finding aligns with Gökırmaklı et al. (2023), who reported that fermentation remained weak in a simple sugary water medium but that ethanol and acetic acid production increased exponentially in nutrient‐rich media such as fig‐based substrates. In the present study, increased sugar content, combined with the nutrients provided by the hazelnut beverage, may have supported microbial metabolic activity, including ethanol production by yeasts and acetic acid formation by *Lactobacillus* and AAB. Similarly, Patel et al. (2022) reported that ethanol and acetic acid were the dominant metabolites during fermentation, increasing from 0.32 to 1.06 g/L and from 3.34 to 11.77 g/L, respectively. Studies suggest that acetic acid contributes to sour, sharp and slightly fruity sensory notes, while ethanol plays a key role in its unique aroma profile (Bozkir et al. 2024; Laureys et al. 2021). In addition, 2‐propanol was identified as a significant alcohol in the samples, however, its concentration tended to decrease with sugar addition. 2‐Propanol is typically associated with solvent‐like odor characteristics and is formed through the reduction of ketones or the metabolism of certain amino acids. An increase in available carbon sources may shift microbial metabolism toward ethanol production, thereby limiting the formation of such secondary alcohols (Laureys and De Vuyst 2014). Consistently, the absence of detectable levels of 2‐pentanol and 1‐butanol in the 5% sugar sample can be attributed to a metabolic redirection under high‐sugar conditions, where fermentation flux is preferentially channeled towards ethanol production. As evidenced by the table, ethanol reached its maximum level (16.36%) in the sample, suggesting an intensified glycolytic flux toward alcoholic fermentation. This shift likely limits the availability of precursor metabolites involved in higher alcohol formation via the Ehrlich pathway, resulting in concentrations falling below the GC–MS detection threshold.

**TABLE 3:** Volatile flavor substances in hazelnut‐based water kefir samples.

			Relative content (%)			
No.	RT	Components	WK‐C	WK‐2.5%	WK‐5%	WK‐7.5%
**Alcohols**						
1	3.15	2‐Propanol	1.91 ± 0.32	0.92 ± 0.92	0.94 ± 0.87	0.89 ± 0.03
2	4.21	Ethanol	6.72 ± 0.10	12.51 ± 1.20	16.36 ± 0.98	15.51 ± 0.44
3	6.56	1‐Propanol, 2‐methyl‐	0.10 ± 0.01	0.12 ± 0.01	0.21 ± 0.10	0.11 ± 0.01
4	7.04	2‐Pentanol	0.10 ± 0.01	0.09 ± 0.01	ND	0.04 ± 0.04
5	7.51	1‐Butanol	0.20 ± 0.01	0.04 ± 0.04	ND	0.02 ± 0.02
6	9.56	1‐Pentanol	0.40 ± 0.04	0.20 ± 0.01	0.08 ± 0.08	0.14 ± 0.06
7	11.92	Hexanol <n‐>	2.88 ± 0.27	2.73 ± 0.37	2.15 ± 0.63	2.68 ± 0.66
8	15.33	Heptanol <n‐>	0.50 ± 0.04	0.32 ± 0.03	0.27 ± 0.06	0.25 ± 0.13
9	18.63	2,3‐Butanediol	2.78 ± 1.12	3.50 ± 0.54	4.06 ± 0.21	4.33 ± 0.48
10	19.16	Capryl alcohol	0.30 ± 0.13	0.51 ± 0.10	0.29 ± 0.07	0.28 ± 0.22
11	20.71	3‐Cyclohexen‐1‐ol, 4‐methyl‐1‐(1‐methylethyl)‐	0.57 ± 0.06	0.65 ± 0.03	0.13 ± 0.13	0.39 ± 0.26
12	22.72	1‐Nonanol	0.44 ± 0.08	0.36 ± 0.01	0.28 ± 0.10	0.23 ± 0.13
13	24.88	1‐Propanol, 3‐(methylthio)‐	ND	0.20 ± 0.05	0.19 ± 0.13	0.17 ± 0.03
14	32.71	Benzeneethanol	0.15 ± 0.02	0.28 ± 0.06	0.59 ± 0.08	0.18 ± 0.10
**Acids**						
15	15.61	Acetic acid	6.29 ± 0.51	10.69 ± 2.85	12.03 ± 1.44	13.88 ± 1.09
16	19.8	Propanoic acid, 2‐methyl‐	0.57 ± 0.05	0.30 ± 0.01	0.32 ± 0.12	0.36 ± 0.09
17	21.89	Butanoic acid	1.22 ± 0.25	2.96 ± 2.13	3.37 ± 0.57	3.82 ± 0.19
18	23.31	iso‐Valeric acid	2.30 ± 0.28	3.82 ± 0.27	3.25 ± 2.25	3.03 ± 0.78
19	30.19	Hexanoic acid	0.47 ± 0.01	0.90 ± 0.38	0.47 ± 0.02	0.46 ± 0.44
20	34.67	Heptanoic acid	ND	0.04 ± 0.04	ND	0.02 ± 0.02
21	39.31	Octanoic acid	0.12 ± 0.03	0.33 ± 0.03	0.31 ± 0.03	0.38 ± 0.15
22	43.91	Pelargonic acid	0.28 ± 0.02	0.55 ± 0.08	0.67 ± 0.02	0.68 ± 0.27
23	48.39	Decanoic acid	ND	0.16 ± 0.04	0.06 ± 0.06	0.11 ± 0.05
24	52.54	Benzoic acid	0.44 ± 0.17	0.39 ± 0.09	0.22 ± 0.07	0.18 ± 0.16
**Aldehydes**						
25	2.65	Acetaldehyde	0.40 ± 0.15	0.34 ± 0.12	0.89 ± 0.71	0.92 ± 0.18
26	3.15	Propanal, 2‐methyl‐	ND	0.03 ± 0.03	0.09 ± 0.01	0.12 ± 0.01
27	3.16	Butanal	ND	0.08 ± 0.01	0.16 ± 0.02	0.21 ± 0.03
28	3.99	Butanal, 2‐methyl‐	ND	ND	0.11 ± 0.11	0.15 ± 0.03
29	4.01	Butanal, 3‐methyl‐	0.27 ± 0.34	0.39 ± 0.23	0.39 ± 0.20	0.41 ± 0.09
30	6.39	Hexanal	0.11 ± 0.03	0.16 ± 0.03	0.19 ± 0.04	0.22 ± 0.06
31	13.2	Nonanal	0.14 ± 0.03	0.09 ± 0.02	0.14 ± 0.05	0.16 ± 0.02
**Ketones**						
32	3.21	2‐Propanone	ND	0.98 ± 0.11	1.07 ± 0.06	1.27 ± 0.18
33	4.78	2,3‐Butanedione	0.41 ± 0.07	0.37 ± 0.06	0.48 ± 0.02	0.55 ± 0.17
34	7.28	3‐Penten‐2‐one, (E)‐	0.29 ± 0.08	0.16 ± 0.01	0.02 ± 0.02	0.09 ± 0.07
37	10.46	2‐Butanone, 3‐hydroxy‐	2.09 ± 0.19	3.50 ± 0.27	3.58 ± 0.43	3.69 ± 0.39
35	15.14	4‐Octanone, 2,3‐epoxy‐2‐methyl‐	0.07 ± 0.07	0.27 ± 0.03	0.29 ± 0.06	0.32 ± 0.05
36	25.37	2‐Cyclohexen‐1‐one, 2‐methyl‐5‐(1‐methylethenyl)‐	0.36 ± 0.02	0.33 ± 0.01	0.38 ± 0.08	0.45 ± 0.13
**Esters**						
38	3.23	Acetic acid, methyl ester	0.34 ± 0.02	0.23 ± 0.03	0.25 ± 0.05	0.29 ± 0.09
39	3.69	Acetic acid, ethyl ester	1.42 ± 0.06	1.65 ± 0.08	1.75 ± 0.71	1.55 ± 0.27
40	4.56	Propanoic acid, 2‐methyl‐, ethyl ester	0.13 ± 0.01	0.13 ± 0.01	ND	ND
41	5.17	Butanoic acid, 2‐methyl‐, methyl ester	0.12 ± 0.02	0.25 ± 0.02	0.28 ± 0.01	0.32 ± 0.12
42	5.84	Butanoic acid, 2‐methyl‐, ethyl ester	1.35 ± 0.34	1.80 ± 0.11	1.99 ± 0.01	1.68 ± 0.21
43	6.1	Butanoic acid, 3‐methyl‐, ethyl ester	1.13 ± 0.01	1.90 ± 0.02	1.43 ± 0.08	0.96 ± 0.19
44	8.72	1‐Butanol, 3‐methyl‐ (impure)	2.22 ± 0.74	6.51 ± 1.81	8.24 ± 0.44	8.58 ± 0.71
45	8.95	Butanoic acid, butyl ester	ND	ND	0.54 ± 0.09	0.49 ± 0.05
46	16.1	Pentanoic acid, 2‐hydroxy‐4‐methyl‐, methyl ester	0.23 ± 0.02	0.36 ± 0.27	0.41 ± 0.05	0.39 ± 0.07
**Others**						
47	8.53	l‐Limonene	0.64 ± 0.17	0.24 ± 0.03	0.26 ± 0.01	0.12 ± 0.12

*Note*: Data is represented as mean ± standard deviation. WK‐C: sugar‐free hazelnut‐based water kefir (Control); WK‐2.5%: hazelnut drink‐based water kefir with 2.5% sugar; WK‐5%: hazelnut drink‐based water kefir with 5% sugar; WK‐7.5%: hazelnut drink‐based water kefir with 7.5% sugar.

Abbreviation: ND, not detected.

Esters contributing to desirable fruity and sweet aromas, such as acetic acid ethyl and methyl esters, and butanoic acid, 2‐methyl‐, ethyl ester, were dominant in all samples. These compounds are typically associated with fruity, sweet, and floral odor descriptors and are considered key aroma‐active volatiles in fermented beverages (Bozkir et al. 2024). Notably, the increase in butanoic acid, 2‐methyl‐, ethyl ester with higher sugar concentrations may indicate enhanced esterification potential under conditions of increased fermentation activity, mediated by yeasts and some LAB. Similarly, butanoic acid butyl ester was detected only in samples with 5% and 7.5% sugar supplementation, suggesting that its formation requires sufficient concentrations of both butanoic acid and butanol substrates. This observation aligns with the understanding that ester synthesis is dependent on the availability of alcohol and acid precursors produced during active fermentation. These esters also have very low odor thresholds, making them highly influential even at low concentrations.

As shown in Table 3, compounds such as 1‐butanol, 3‐methyl‐ (isoamyl alcohol), and 2,3‐butanedione (diacetyl) tended to increase with sugar content, indicating diverse metabolic responses within the water kefir microbiota. These compounds are widely reported as contributors to fermented beverage aroma. Patel et al. (2022) reported that higher alcohols are formed as a result of amino acid metabolism and that yeasts show a positive correlation with the production of these compounds. Similarly, Athanasiadis et al. (2001) found that in fermentations with kefir yeast, increasing sugar concentration led to a general increase in volatile byproducts, with amyl alcohols (a mixture of 2‐methyl‐1‐butanol and 3‐methyl‐1‐butanol) being among the main products. The increase in 2,3‐butanedione content can be attributed to the activity of citrate‐fermenting microorganisms such as *Lactococcus* and *Leuconostoc* spp., which may contribute to buttery aroma notes in fermented beverages (Gökırmaklı et al. 2023).

Among the aldehydes identified in water kefir samples were acetaldehyde, propanal, butanal, and branched‐chain aldehydes such as 2‐methylpropanal and 3‐methylbutanal. Acetaldehyde is particularly important for its contribution to the fruity and fresh aroma characteristics of fermented beverages. The results indicate that acetaldehyde levels increased with sugar addition, likely due to its role as an intermediate in ethanol metabolism. Acetaldehyde produced by yeasts can be reduced to ethanol via alcohol dehydrogenase enzymes, and under certain conditions, it may accumulate in the medium (Marsh et al. 2014). The formation of branched‐chain aldehydes (e.g., 2‐methylpropanal and 3‐methylbutanal) is generally explained via the Ehrlich pathway, which involves the catabolism of branched‐chain amino acids such as leucine, isoleucine, and valine, contributing to malty and fruity aroma notes. Enhanced protein and amino acid metabolism by fermentation may support the formation of these compounds (Hazelwood et al. 2008). Similarly, 2‐methylbutanal, another branched‐chain aldehyde, is derived from the Ehrlich pathway via the catabolism of isoleucine. In the present study, this compound was detected only in samples with 5% and 7.5% sugar supplementation, whereas it remained below the detection limit in the control and 2.5% sugar samples. This observation suggests that a minimum sugar concentration is required to activate amino acid catabolism and provide the necessary energy and redox balance for the Ehrlich pathway to proceed (Hazelwood et al. 2008).

### 3.7 Allergenicity

Food allergies are a major public health concern, affecting 1%–10% of the population, with higher prevalence in children (5%–12.6%) and variation by region (Pi et al. 2022). Eight food groups—including dairy, eggs, fish, shellfish, peanuts, tree nuts, wheat, and soy—are responsible for over 90% of reactions, and hazelnuts have recently been recognized as a priority allergen in the European Union. Hazelnut allergies are common in developed countries and can trigger severe immune responses, including anaphylaxis, skin reactions, and respiratory or digestive issues (Matricardi et al. 2016). Cross‐reactivity with other tree nuts (almonds, walnuts, pistachios) and respiratory allergies to birch, hazelnut, and alder pollens are frequent due to similar protein structures (Camus‐Ela et al. 2025; Huang et al. 2023). Food processing can alter the structure and immunoreactivity of hazelnut proteins. Heat treatments, such as roasting, can either reduce or, in some cases, maintain allergenic potential (Giannetti et al. 2023; Kabasser et al. 2024). In this study, homogenization and pasteurization applied during hazelnut beverage preparation may also have influenced allergenic properties. Processing affects gastrointestinal digestion of allergens, with heat and pressure treatments reducing IgE binding, though not always completely eliminating allergenic potential (Arribas et al. 2024).

**FIGURE 4:**
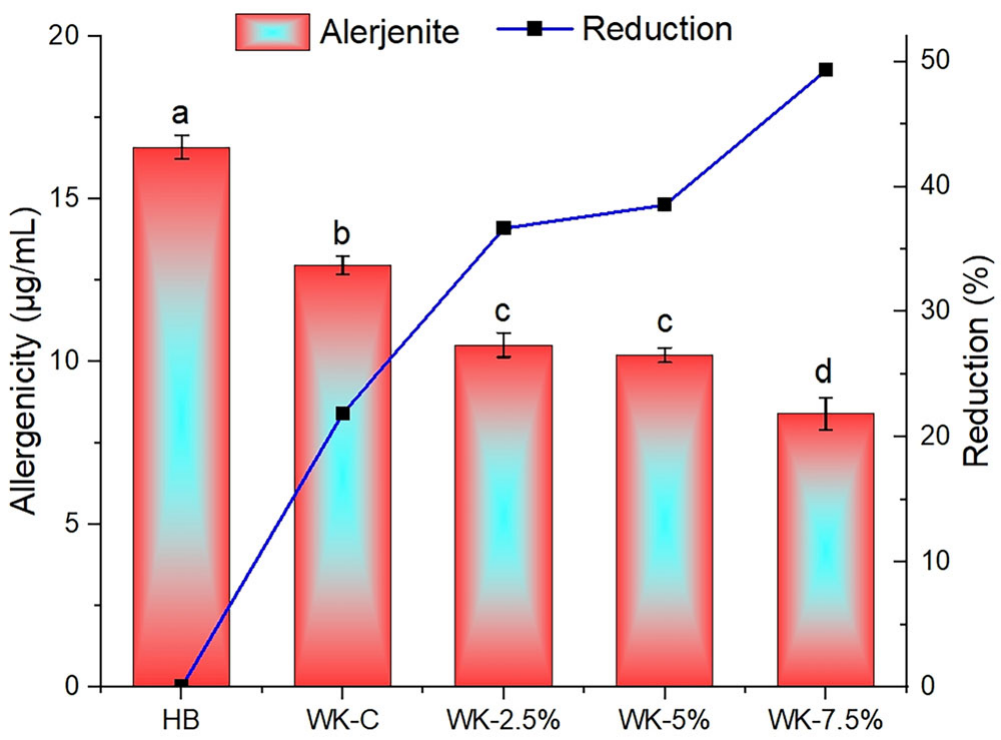
Allergenicity (a) and sensory properties (b) of hazelnut beverage and hazelnut‐based water kefir samples (HB: Hazelnut beverage; WK‐C: sugar‐free hazelnut‐based water kefir [Control]; WK‐2.5%: hazelnut drink‐based water kefir with 2.5% sugar; WK‐5%: hazelnut drink‐based water kefir with 5% sugar; WK‐7.5%: hazelnut drink‐based water kefir with 7.5% sugar).

In the present study, the allergen level in the hazelnut beverage used for kefir production was 16.6 µg/mL (Figure 4). Fermentation reduced allergen levels, with hazelnut‐based kefir samples ranging from 8.42 to 12.97 µg/mL. In the sugar‐free control kefir sample, a 21.86% reduction in allergenicity was observed due to fermentation. Similar results were reported by Erem and Kilic‐Akyilmaz (2024), who found that fermentation reduced allergenicity by approximately 72% in samples prepared from roasted hazelnuts and by approximately 70% in samples from raw hazelnuts. Similarly, Tang et al. (2023) demonstrated that the immunogenicity of apricot kernels decreased by 29.64% after fermentation with *Lactobacillus plantarum*, attributing this reduction to protease activity. Mattison et al. (2020) reported that cashew allergenicity was significantly reduced by fermentation, with IgE binding levels decreasing from 100% to 20%. In another study by J. M. Chen et al. (2020), a substantial decrease in cashew allergen content was observed when comparing the pre‐fermentation stage (0 h) with the final product stage (72 h). The lower reduction observed in this study compared to the literature may be attributed to the complex composition of the hazelnut beverage matrix, where protein–polyphenol interactions can limit the accessibility of allergenic proteins to microbial enzymes. In addition, differences in fermentation conditions and microbiota composition may have further restricted proteolytic breakdown, resulting in a more moderate reduction in allergenicity.

Fermentation is considered an effective approach for reducing food allergenicity, as it can decrease both the amount and immunoreactivity of allergenic proteins, potentially alleviating allergic responses in sensitive individuals. During fermentation, allergen reduction mainly occurs through proteolysis and acid‐induced denaturation, which can degrade proteins and alter epitope structures (Rui et al. 2019). In addition, glycosylation and Maillard reaction–related modifications may further reduce allergenicity by masking or modifying IgE‐binding sites. Moreover, some allergens may partition into the aqueous phase during fermentation, contributing to a reduction in their detectable levels in the final product (Pi et al. 2019). Among these mechanisms, proteolysis and acid‐induced denaturation are generally considered the primary contributors to allergen reduction in fermented foods (Pi et al. 2022).

In the present study, sugar addition during fermentation was associated with a further reduction in measured allergen levels compared to the control sample. In samples with low‐, medium‐, and high‐sugar concentrations, the reduction rates ranged from 36.65% to 49.31%, all exceeding the reduction observed in the control. While no statistically significant difference was observed between samples containing low and moderate sugar levels, the sample with 7.5% added sugar exhibited a more pronounced reduction in allergenicity than the others. This trend may be related to enhanced microbial growth and metabolic activity under higher sugar conditions, which can influence acidification and proteolytic activity during fermentation. Increased acidity and microbial activity may have contributed indirectly to protein modification and degradation. The observed microbial counts and pH changes in the samples support this interpretation.

## 4 Conclusion

This study demonstrated that hazelnut cake, a low‐value by‐product of the cold‐press oil industry, can be effectively utilized for the production of a hazelnut‐based water kefir beverage.

Sugar supplementation significantly influenced fermentation performance by enhancing microbial growth and metabolic activity, which in turn affected the physicochemical, rheological, and bioactive properties of the final product. Among the tested formulations, 5% sugar provided the most balanced improvement in WHC and physical stability, while higher sugar levels promoted increased viscosity and structural strength due to intensified microbial metabolite production and higher total solids. All samples exhibited pseudoplastic behavior, consistent with typical fermented beverages. Fermentation also contributed to increased total phenolic content and antioxidant activity, likely associated with microbial enzymatic activity and the release of bound phenolic compounds. In addition, GC–MS analysis revealed that sugar concentration modulated the volatile profile, particularly enhancing ethanol, acetic acid, and fruity esters, which are important contributors to aroma complexity. Importantly, fermentation reduced hazelnut allergenicity across all samples, highlighting its potential to improve product safety. Overall, hazelnut‐based water kefir represents a promising plant‐based functional beverage derived from an agro‐industrial by‐product. Further research is needed to fully understand its fermentation dynamics, Maillard reactions, physicochemical changes, and storage stability to ensure product quality, safety, and optimal shelf life.

## Author Contributions

**Latife Betul Gul**: formal analysis, investigation, methodology, writing – review and editing. **Abdullah Akgun**: conceptualization, methodology, investigation, formal analysis, writing – review and editing. **Osman Gul**: supervision, visualization, validation, methodology, investigation, formal analysis, data curation, conceptualization, writing – review and editing, writing – original draft.

## Conflicts of Interest

The authors declare no conflicts of interest.

## Data Availability

Data will be made available on request.

## References

[jfds71432-bib-0001] Abdul, A. , S. Kumaji , and F. Duengo . 2018. “Pengaruh Penambahan Susu Sapi Terhadap Kadar Asam Laktat Pada Pembuatan Yoghurt Jagung Manis Oleh Streptococcus Thermophillus Dan Lactobacillus Bulgaricus.” Bioma: Jurnal Biologi Makassar 3, no. 2: 1–9.

[jfds71432-bib-0002] Acker, W. W. , J. M. Plasek , K. G. Blumenthal , et al. 2017. “Prevalence of Food Allergies and Intolerances Documented in Electronic Health Records.” Journal of Allergy and Clinical Immunology 140, no. 6: 1587–1591.e1. 10.1016/j.jaci.2017.04.006. 28577971 PMC7059078

[jfds71432-bib-0003] Alasalvar, C. , J. S. Amaral , G. Satır , and F. Shahidi . 2009. “Lipid Characteristics and Essential Minerals of Native Turkish Hazelnut Varieties (*Corylus avellana* L.).” Food Chemistry 113, no. 4: 919–925. 10.1016/j.foodchem.2008.08.019.

[jfds71432-bib-0004] Alrosan, M. , T. C. Tan , A. M. Easa , et al. 2023. “Evaluation of Quality and Protein Structure of Natural Water Kefir‐Fermented Quinoa Protein Concentrates.” Food Chemistry 404, no. Pt B: 134614. 10.1016/j.foodchem.2022.134614. 36444092

[jfds71432-bib-0005] Amaretti, A. , M. Di Nunzio , A. Pompei , S. Raimondi , M. Rossi , and A. Bordoni . 2013. “Antioxidant Properties of Potentially Probiotic Bacteria: In Vitro and In Vivo Activities.” Applied Microbiology and Biotechnology 97, no. 2: 809–817. 10.1007/s00253-012-4241-7. 22790540

[jfds71432-bib-0006] AOAC . 2000. Official Methods of Analysis. 17th ed. AOAC.

[jfds71432-bib-0007] Araújo, C. d. S. , L. L. Macedo , and L. J. Q. Teixeira . 2023. “Use of Mid‐Infrared Spectroscopy to Predict the Content of Bioactive Compounds of a New Non‐Dairy Beverage Fermented With Water Kefir.” LWT 176: 114514. 10.1016/j.lwt.2023.114514.

[jfds71432-bib-0008] Arribas, C. , A. Sanchiz , M. M. Pedrosa , S. Perez‐Garcia , R. Linacero , and C. Cuadrado . 2024. “Impact of Heat and Pressure Processing Treatments on the Digestibility of Peanut, Hazelnut, Pistachio and Cashew Allergens.” Foods 13, no. 22: 3549. 10.3390/foods13223549. 39593965 PMC11593142

[jfds71432-bib-0009] Atalar, I. 2019. “Functional Kefir Production From High Pressure Homogenized Hazelnut Milk.” LWT 107: 256–263. 10.1016/j.lwt.2019.03.013.

[jfds71432-bib-0010] Atalar, I. , A. Kurt , O. Gul , and F. Yazici . 2021. “Improved Physicochemical, Rheological and Bioactive Properties of Ice Cream: Enrichment With High Pressure Homogenized Hazelnut Milk.” International Journal of Gastronomy and Food Science 24: 100358. 10.1016/j.ijgfs.2021.100358.

[jfds71432-bib-0011] Athanasiadis, I. , D. Boskou , M. Kanellaki , and A. A. Koutinas . 2001. “Effect of Carbohydrate Substrate on Fermentation by Kefir Yeast Supported on Delignified Cellulosic Materials.” Journal of Agricultural and Food Chemistry 49, no. 2: 658–663. 10.1021/jf0006628. 11262008

[jfds71432-bib-0012] Atik, D. S. , T. Demirci , H. İ. Öztürk , S. Demirci , D. Sert , and N. Akın . 2020. “Chia Seed Mucilage versus Guar Gum: Effects on Microstructural, Textural, and Antioxidative Properties of Set‐Type Yoghurts.” Brazilian Archives of Biology and Technology 63: e20190702. 10.1590/1678-4324-2020190702.

[jfds71432-bib-0013] Azi, F. , C. Tu , H. A. Rasheed , and M. Dong . 2020. “Comparative Study of the Phenolics, Antioxidant and Metagenomic Composition of Novel Soy Whey‐Based Beverages Produced Using Three Different Water Kefir Microbiota.” International Journal of Food Science & Technology 55, no. 4: 1689–1697. 10.1111/ijfs.14439.

[jfds71432-bib-0014] Bernat, N. , M. Cháfer , C. González‐Martínez , J. Rodríguez‐García , and A. Chiralt . 2015. “Optimisation of Oat Milk Formulation to Obtain Fermented Derivatives by Using Probiotic Lactobacillus Reuteri Microorganisms.” Food Science and Technology International 21, no. 2: 145–157. 10.1177/1082013213518936. 24464238

[jfds71432-bib-0015] Bozkir, E. , B. Yilmaz , H. Sharma , T. Esatbeyoglu , and F. Ozogul . 2024. “Challenges in Water Kefir Production and Limitations in human Consumption: A Comprehensive Review of Current Knowledge.” Heliyon 10, no. 13: e33501. 10.1016/j.heliyon.2024.e33501. 39035485 PMC11259891

[jfds71432-bib-0016] Bueno, R. S. , J. B. Ressutte , N. N. Y. Hata , et al. 2021. “Quality and Shelf Life Assessment of a New Beverage Produced From Water Kefir Grains and Red Pitaya.” LWT 140: 110770. 10.1016/j.lwt.2020.110770.

[jfds71432-bib-0017] Camus‐Ela, M. , Y. Wang , G. H. Rennie , V. Raghavan , and J. Wang . 2025. “Update on Hazelnut Allergy: Allergen Characterization, Epidemiology, Food Processing Technique and Detecting Strategy.” Comprehensive Reviews in Food Science and Food Safety 24, no. 2: e70098. 10.1111/1541-4337.70098. 39898897 PMC11789833

[jfds71432-bib-0018] Chen, J. M. , K. F. Al , L. J. Craven , et al. 2020. “Nutritional, Microbial, and Allergenic Changes During the Fermentation of Cashew ‘Cheese’ Product Using a Quinoa‐Based Rejuvelac Starter Culture.” Nutrients 12, no. 3: 648. 10.3390/nu12030648. 32121191 PMC7146175

[jfds71432-bib-0019] Chen, L. , P. Song , F. Jia , and J. S. Wang . 2012. “Reducing the Allergenicity From Food by Microbial Fermentation.” Advanced Materials Research 524–527: 2302–2305. 10.4028/www.scientific.net/AMR.524-527.2302.

[jfds71432-bib-0020] Comak Gocer, E. M. , and E. Koptagel . 2023. “Production and Evaluation of Microbiological & Rheological Characteristics of Kefir Beverages Made From Nuts.” Food Bioscience 52: 102367. 10.1016/j.fbio.2023.102367.

[jfds71432-bib-0021] Corona, O. , W. Randazzo , A. Miceli , et al. 2016. “Characterization of Kefir‐Like Beverages Produced From Vegetable Juices.” LWT—Food Science and Technology 66: 572–581. 10.1016/j.lwt.2015.11.014.

[jfds71432-bib-0022] Cufaoglu, G. , and A. N. Erdinc . 2023a. “An Alternative Source of Probiotics: Water Kefir.” Food Frontiers 4, no. 1: 21–31. 10.1002/fft2.200.

[jfds71432-bib-0023] Cufaoglu, G. , and A. N. Erdinc . 2023b. “Comparative Analyses of Milk and Water Kefir: Fermentation Temperature, Physicochemical Properties, Sensory Qualities, and Metagenomic Composition.” Food Bioscience 55: 103079. 10.1016/j.fbio.2023.103079.

[jfds71432-bib-0024] Dickinson, J. R. , and A. L. Kruckeberg . 2006. “Carbohydrate Metabolism.” In Yeasts in Food and Beverages, 215–242. Springer Berlin Heidelberg.

[jfds71432-bib-0025] Dikmetas, D. N. , E. G. Acar , F. D. Ceylan , F. İlkadım , H. Özer , and F. Karbancioglu‐Guler . 2026. “Functional Fermented Fruit Juice Production and Characterization by Using Water Kefir Grains.” Journal of Food Science and Technology 63, no. 2: 378–391. 10.1007/s13197-025-06209-y. 41737712 PMC12926261

[jfds71432-bib-0026] El‐Ghaish, S. , A. Ahmadova , I. Hadji‐Sfaxi , et al. 2011. “Potential Use of Lactic Acid Bacteria for Reduction of Allergenicity and for Longer Conservation of Fermented Foods.” Trends in Food Science & Technology 22, no. 9: 509–516. 10.1016/j.tifs.2011.05.003.

[jfds71432-bib-0027] Erem, E. , and M. Kilic‐Akyilmaz . 2024. “Development of a Stable Fermented Creamy Structure From Hazelnut in the Scope of Plant‐Based Food Production.” Food and Bioproducts Processing 145: 128–135. 10.1016/j.fbp.2024.03.006.

[jfds71432-bib-0028] Erem, E. , B. Sezer , F. Ortakci , and M. Kilic‐Akyilmaz . 2026. “Bioactivity Potential of Hazelnut‐Based Product Fermented With Different Cultures of Lactic Acid Bacteria.” Biofactors 52, no. 1: e70075. 10.1002/biof.70075. 41545202

[jfds71432-bib-0029] Esatbeyoglu, T. , A. Fischer , A. D. S. Legler , et al. 2023. “Physical, Chemical, and Sensory Properties of Water Kefir Produced From Aronia Melanocarpa Juice and Pomace.” Food Chemistry: X 18: 100683. 10.1016/j.fochx.2023.100683. 37138825 PMC10149414

[jfds71432-bib-0030] Fels, L. , F. Jakob , R. F. Vogel , and D. Wefers . 2018. “Structural Characterization of the Exopolysaccharides From Water Kefir.” Carbohydrate Polymers 189: 296–303. 10.1016/j.carbpol.2018.02.037. 29580412

[jfds71432-bib-0031] Fiorda, F. A. , G. V. de Melo Pereira , V. Thomaz‐Soccol , et al. 2017. “Microbiological, Biochemical, and Functional Aspects of Sugary Kefir Fermentation—A Review.” Food Microbiology 66: 86–95. 10.1016/j.fm.2017.04.004. 28576377

[jfds71432-bib-0032] Fu, W. , W. Xue , C. Liu , Y. Tian , K. Zhang , and Z. Zhu . 2020. “Screening of Lactic Acid Bacteria and Yeasts From Sourdough as Starter Cultures for Reduced Allergenicity Wheat Products.” Foods 9, no. 6: 751. 10.3390/foods9060751. 32517155 PMC7353608

[jfds71432-bib-0033] Giannetti, A. , A. Ruggi , G. Ricci , G. Gianni , and C. Caffarelli . 2023. “Natural History of Hazelnut Allergy and Current Approach to Its Diagnosis and Treatment.” Children 10, no. 3: 585. 10.3390/children10030585. 36980143 PMC10047188

[jfds71432-bib-0034] Gökırmaklı, Ç. , İ. Gün , M. O. Kartal , and Z. B. Güzel‐Seydim . 2025. “Antioxidant Capacity, Volatile Compounds, Microbial, Chemical and Sensory Properties of Plum (*Prunus domestica*) Juice Water Kefir.” Discover Food 5, no. 1: 33. 10.1007/s44187-025-00297-7.

[jfds71432-bib-0035] Gökırmaklı, Ç. , and Z. B. Güzel‐Seydim . 2022. “Water Kefir Grains vs. Milk Kefir Grains: Physical, Microbial and Chemical Comparison.” Journal of Applied Microbiology 132, no. 6: 4349–4358. 10.1111/jam.15532. 35301787

[jfds71432-bib-0036] Gökırmaklı, Ç. , Y. K. Yüceer , and Z. B. Guzel‐Seydim . 2023. “Chemical, Microbial, and Volatile Changes of Water Kefir During Fermentation With Economic Substrates.” European Food Research and Technology 249, no. 7: 1717–1728. 10.1007/s00217-023-04242-9.

[jfds71432-bib-0037] Gul, O. , I. Atalar , M. Mortas , et al. 2022. “Potential Use of High Pressure Homogenized Hazelnut Beverage for a Functional Yoghurt‐Like Product.” Anais da Academia Brasileira de Ciências 94, no. 1: e20191172. 10.1590/0001-3765202220191172. 35107513

[jfds71432-bib-0038] Gul, O. , I. Atalar , M. Mortas , F. T. Saricaoglu , and F. Yazıcı . 2018a. “Application of TOPSIS Methodology to Determine Optimum Hazelnut Cake Concentration and High Pressure Homogenization Condition for Hazelnut Milk Production Based on Physicochemical, Structural and Sensory Properties.” Journal of Food Measurement and Characterization 12, no. 4: 2404–2415. 10.1007/s11694-018-9857-6.

[jfds71432-bib-0039] Gul, O. , I. Atalar , F. T. Saricaoglu , and F. Yazici . 2018. “Effect of Multi‐Pass High Pressure Homogenization on Physicochemical Properties of Hazelnut Milk From Hazelnut Cake: An Investigation by Response Surface Methodology.” Journal of Food Processing and Preservation 42, no. 5: e13615. 10.1111/jfpp.13615.

[jfds71432-bib-0040] Gül, L. B. , S. Bekbay , A. Akgün , and O. Gül . 2023. “Effect of Oleaster (*Elaeagnus angustifolia* L.) Flour Addition Combined With High‐Pressure Homogenization on the Acidification Kinetics, Physicochemical, Functional, and Rheological Properties of Kefir.” Food Science & Nutrition 11, no. 9: 5325–5337. 10.1002/fsn3.3491. 37701222 PMC10494617

[jfds71432-bib-0041] Gul, O. , F. T. Saricaoglu , and I. Atalar . 2021. “Effect of High Pressure Homogenization on Microstructure and Rheological Properties of Hazelnut Beverage Cold‐Set Gels Induced Glucono‐δ‐Lactone.” LWT 143: 111154. 10.1016/j.lwt.2021.111154.

[jfds71432-bib-0042] Gul, O. , F. T. Saricaoglu , M. Mortas , I. Atalar , and F. Yazici . 2017. “Effect of High Pressure Homogenization (HPH) on Microstructure and Rheological Properties of Hazelnut Milk.” Innovative Food Science & Emerging Technologies 41: 411–420. 10.1016/j.ifset.2017.05.002.

[jfds71432-bib-0043] Gülhan, M. F. , A. Gülhan , and C. Düşgün . 2025. “Physico‐Chemical and Microbiological Properties of Water Kefir Produced From Carob (*Ceratonia siliqua* L.) Sherbet.” Food Science and Biotechnology 34, no. 1: 103–114. 10.1007/s10068-024-01682-1. 39758735 PMC11695531

[jfds71432-bib-0044] Gulitz, A. , J. Stadie , M. Wenning , M. A. Ehrmann , and R. F. Vogel . 2011. “The Microbial Diversity of Water Kefir.” International Journal of Food Microbiology 151, no. 3: 284–288. 10.1016/j.ijfoodmicro.2011.09.016. 22000549

[jfds71432-bib-0045] Hastrawirid, R. W. , B. Dwiloka , and H. Rizqiati . 2024. “Effect of Sucrose Addition on Viscosity, pH, Total Yeast, and Hedonic Quality of Water Kefir Roselle.” Journal of Applied Food Technology 11, no. 2: 61–65. 10.17728/jaft.22719.

[jfds71432-bib-0046] Hazelwood, L. A. , J. M. Daran , A. J. van Maris , J. T. Pronk , and J. R. Dickinson . 2008. “The Ehrlich Pathway for Fusel Alcohol Production: A Century of Research on Saccharomyces Cerevisiae Metabolism.” Applied and Environmental Microbiology 74, no. 8: 2259–2266. 10.1128/AEM.02625-07. 18281432 PMC2293160

[jfds71432-bib-0047] Huang, Y. Y. , Y. T. Liang , J. M. Wu , et al. 2023. “Advances in the Study of Probiotics for Immunomodulation and Intervention in Food Allergy.” Molecules 28, no. 3: 1242. 10.3390/molecules28031242. 36770908 PMC9919562

[jfds71432-bib-0048] Kabasser, S. , C. Radauer , E. Eber , et al. 2024. “Cosensitization to the 3 Nonhomologous Major Cashew Allergens Ana o 1, Ana o 2, and Ana o 3 Is Caused by IgE Cross‐Reactivity.” Journal of Investigational Allergology & Clinical Immunology 34, no. 1: 38–48. 10.18176/jiaci.0867. 36331131

[jfds71432-bib-0049] Kitchen, B. , and G. Williamson . 2024. “Melanoidins and (poly)Phenols: An Analytical Paradox.” Current Opinion in Food Science 60: 101217. 10.1016/j.cofs.2024.101217.

[jfds71432-bib-0050] Koh, W. Y. , U. Utra , A. Rosma , M. E. Effarizah , W. I. W. Rosli , and Y. H. Park . 2018. “Development of a Novel Fermented Pumpkin‐Based Beverage Inoculated With Water Kefir Grains: A Response Surface Methodology Approach.” Food Science and Biotechnology 27, no. 2: 525–535. 10.1007/s10068-017-0245-5. 30263777 PMC6049631

[jfds71432-bib-0051] La Torre, C. , R. Pino , A. Fazio , P. Plastina , and M. R. Loizzo . 2025. “Synergistic Bioactive Potential of Combined Fermented Kombucha and Water Kefir.” Beverages 11, no. 3: 65. 10.3390/beverages11030065.

[jfds71432-bib-0052] Laureys, D. , and L. De Vuyst . 2014. “Microbial Species Diversity, Community Dynamics, and Metabolite Kinetics of Water Kefir Fermentation.” Applied and Environmental Microbiology 80, no. 8: 2564–2572. 10.1128/AEM.03978-13. 24532061 PMC3993195

[jfds71432-bib-0053] Laureys, D. , F. Leroy , T. Hauffman , et al. 2021. “The Type and Concentration of Inoculum and Substrate as Well as the Presence of Oxygen Impact the Water Kefir Fermentation Process.” Frontiers in Microbiology 12: 628599. 10.3389/fmicb.2021.628599. 33643256 PMC7904701

[jfds71432-bib-0054] Limbad, M. , N. Gutierrez‐Maddox , N. Hamid , K. Kantono , T. Liu , and T. Young . 2023. “Microbial and Chemical Changes During Fermentation of Coconut Water Kefir Beverage.” Applied Sciences 13, no. 12: 7257. 10.3390/app13127257.

[jfds71432-bib-0055] Lynch, K. M. , S. Wilkinson , L. Daenen , and E. K. Arendt . 2021. “An Update on Water Kefir: Microbiology, Composition and Production.” International Journal of Food Microbiology 345: 109128. 10.1016/j.ijfoodmicro.2021.109128. 33751986

[jfds71432-bib-0056] Magalhães, K. T. , G. Dragone , G. V. de Melo Pereira , et al. 2011. “Comparative Study of the Biochemical Changes and Volatile Compound Formations During the Production of Novel Whey‐Based Kefir Beverages and Traditional Milk Kefir.” Food Chemistry 126, no. 1: 249–253. 10.1016/j.foodchem.2010.11.012.

[jfds71432-bib-0057] Marsh, A. J. , O. O'Sullivan , C. Hill , R. P. Ross , and P. D. Cotter . 2014. “Sequence‐Based Analysis of the Bacterial and Fungal Compositions of Multiple Kombucha (Tea Fungus) Samples.” Food Microbiology 38: 171–178. 10.1016/j.fm.2013.09.003. 24290641

[jfds71432-bib-0058] Martínez‐Torres, A. , S. Gutiérrez‐Ambrocio , P. Heredia‐del‐Orbe , L. Villa‐Tanaca , and C. Hernández‐Rodríguez . 2017. “Inferring the Role of Microorganisms in Water Kefir Fermentations.” International Journal of Food Science & Technology 52, no. 2: 559–571. 10.1111/ijfs.13312.

[jfds71432-bib-0059] Matricardi, P. M. , J. Kleine‐Tebbe , H. J. Hoffmann , et al. 2016. “EAACI Molecular Allergology User's Guide.” Pediatric Allergy and Immunology 27, no. S23: 1–250. 10.1111/pai.12563. 27288833

[jfds71432-bib-0060] Mattison, C. P. , K. J. Aryana , K. Clermont , et al. 2020. “Microbiological, Physicochemical, and Immunological Analysis of a Commercial Cashew Nut‐Based Yogurt.” International Journal of Molecular Sciences 21, no. 21: 8267. 10.3390/ijms21218267. 33158240 PMC7663355

[jfds71432-bib-0061] Michlmayr, H. , and W. Kneifel . 2014. “β‐Glucosidase Activities of Lactic Acid Bacteria: Mechanisms, Impact on Fermented Food and Human Health.” FEMS Microbiology Letters 352, no. 1: 1–10. 10.1111/1574-6968.12348. 24330034

[jfds71432-bib-0062] Mitra, S. , and B. C. Ghosh . 2019. “Quality Characteristics of Kefir as a Carrier for Probiotic Lactobacillus Rhamnosus GG.” International Journal of Dairy Technology 73, no. 2: 384–391. 10.1111/1471-0307.12664.

[jfds71432-bib-0063] Mohan, A. , J. Hadi , N. Gutierrez‐Maddox , et al. 2020. “Sensory, Microbiological and Physicochemical Characterisation of Functional Manuka Honey Yogurts Containing Probiotic Lactobacillus Reuteri DPC16.” Foods 9, no. 1: 106. 10.3390/foods9010106. 31963907 PMC7023061

[jfds71432-bib-0064] Mu, G. , H. Li , Y. Tuo , Y. Gao , and Y. Zhang . 2019. “Antioxidative Effect of *Lactobacillus plantarum* Y44 on 2,2′‐Azobis(2‐Methylpropionamidine) Dihydrochloride (ABAP)‐Damaged Caco‐2 Cells.” Journal of Dairy Science 102, no. 8: 6863–6875. 10.3168/jds.2019-16447. 31178173

[jfds71432-bib-0065] Nascimento, R. Q. , K. M. Deamici , P. P. L. G. Tavares , et al. 2022. “Improving Water Kefir Nutritional Quality via Addition of Viable Spirulina Biomass.” Bioresource Technology Reports 17: 100914. 10.1016/j.biteb.2021.100914.

[jfds71432-bib-0066] Nguyen, N. T. , N. P. Nguyen , L. T. K. Vu , and N. L. Le . 2025. “Water Kefir From Acerola‐Carrot Blends: Microbial, Antioxidant, Volatile, and Sensory Properties.” Journal of Food Measurement and Characterization 20: 3307–3317. 10.1007/s11694-025-03922-2.

[jfds71432-bib-0067] Öztürk, H. İ. , S. Aydın , D. Sözeri , T. Demirci , D. Sert , and N. Akın . 2018. “Fortification of Set‐Type Yoghurts With *Elaeagnus angustifolia* L. Flours: Effects on Physicochemical, Textural, and Microstructural Characteristics.” LWT 90: 620–626. 10.1016/j.lwt.2018.01.012.

[jfds71432-bib-0068] Ozturkoglu‐Budak, S. , C. Akal , and A. Yetisemiyen . 2016. “Effect of Dried Nut Fortification on Functional, Physicochemical, Textural, and Microbiological Properties of Yogurt.” Journal of Dairy Science 99, no. 11: 8511–8523. 10.3168/jds.2016-11217. 27638255

[jfds71432-bib-0069] Palacios‐Castillo, A. E. , T. N. Campoverde‐Quilca , J. Núñez‐Pérez , et al. 2025. “Optimising a Functional Beverage From Quinoa and Cherimoya Mixtures Fermented With Water Kefir Grains.” Foods 14, no. 20: 3464. 10.3390/foods14203464. 41153999 PMC12562328

[jfds71432-bib-0070] Papadopoulou, D. , V. Chrysikopoulou , A. Rampaouni , et al. 2025. “Antioxidant, Antithrombotic and Anti‐Inflammatory Properties of Amphiphilic Bioactives From Water Kefir Grains and Its Apple Pomace‐Based Fermented Beverage.” Antioxidants 14, no. 2: 164. 10.3390/antiox14020164. 40002351 PMC11851739

[jfds71432-bib-0071] Paredes, J. L. , M. L. Escudero‐Gilete , and I. M. Vicario . 2022. “A New Functional Kefir Fermented Beverage Obtained From Fruit and Vegetable Juice: Development and Characterization.” LWT 154: 112728. 10.1016/j.lwt.2021.112728.

[jfds71432-bib-0072] Patel, S. H. , J. P. Tan , R. A. Börner , et al. 2022. “A Temporal View of the Water Kefir Microbiota and Flavour Attributes.” Innovative Food Science & Emerging Technologies 80: 103084. 10.1016/j.ifset.2022.103084.

[jfds71432-bib-0073] Patra, T. , C. Axel , Å. Rinnan , and K. Olsen . 2022. “The Physicochemical Stability of Oat‐Based Drinks.” Journal of Cereal Science 104: 103422. 10.1016/j.jcs.2022.103422.

[jfds71432-bib-0074] Pi, X. , B. Dong , X. Wu , et al. 2020. “Effects of Liquid Fermentation by Bacillus Natto on the Physicochemical Properties of Peanut and Allergenicity of Peanut Protein During Digestion.” Science and Technology of Food Industry 41, no. 8: 49–61.

[jfds71432-bib-0075] Pi, X. , Y. Wan , Y. Yang , et al. 2019. “Research Progress in Peanut Allergens and Their Allergenicity Reduction.” Trends in Food Science & Technology 93: 212–220. 10.1016/j.tifs.2019.09.014.

[jfds71432-bib-0076] Pi, X. , Y. Yang , Y. Sun , et al. 2022. “Recent Advances in Alleviating Food Allergenicity Through Fermentation.” Critical Reviews in Food Science and Nutrition 62, no. 26: 7255–7268. 10.1080/10408398.2021.1913093. 33951963

[jfds71432-bib-0077] Pinto, L. C. , T. P. Oliveira , R. Souza , et al. 2020. “Probiotic Kefir‐Fermented Beverage‐Based *Colocasia esculenta* L.: Development, Characterization, and Microbiological Stability During Chilled Storage.” Journal of Food Processing and Preservation 45, no. 2: e15113. 10.1111/jfpp.15113.

[jfds71432-bib-0078] Pisoschi, A. M. , and A. Pop . 2015. “The Role of Antioxidants in the Chemistry of Oxidative Stress: A Review.” European Journal of Medicinal Chemistry 97: 55–74. 10.1016/j.ejmech.2015.04.040. 25942353

[jfds71432-bib-0079] Prado, M. R. , L. M. Blandón , L. P. S. Vandenberghe , et al. 2015. “Milk Kefir: Composition, Microbial Cultures, Biological Activities, and Related Products.” Frontiers in Microbiology 6: 1177. 10.3389/fmicb.2015.01177. 26579086 PMC4626640

[jfds71432-bib-0080] Rejdlová, A. , R. N. Salek , Z. Míšková , et al. 2023. “Physical Characterization of a Novel Carrot Juice Whey‐enriched Beverage Fermented With Milk or Water Kefir Starter Cultures.” Foods 12, no. 18: 3368. 10.3390/foods12183368. 37761077 PMC10528688

[jfds71432-bib-0081] Rui, X. , J. Huang , G. Xing , Q. Zhang , W. Li , and M. Dong . 2019. “Changes in Soy Protein Immunoglobulin E Reactivity, Protein Degradation, and Conformation Through Fermentation With *Lactobacillus plantarum* Strains.” LWT 99: 156–165. 10.1016/j.lwt.2018.09.034.

[jfds71432-bib-0082] Sadiq Butt, M. , M. Tahir‐Nadeem , M. K. Khan , R. Shabir , and M. S. Butt . 2008. “Oat: Unique Among the Cereals.” European Journal of Nutrition 47, no. 2: 68–79. 10.1007/s00394-008-0698-7. 18301937

[jfds71432-bib-0083] Şafak, H. , I. Gün , M. Tudor Kalit , and S. Kalit . 2023. “Physico‐Chemical, Microbiological and Sensory Properties of Water Kefir Drinks Produced From Demineralized Whey and Dimrit and Shiraz Grape Varieties.” Foods 12, no. 9: 1851. 10.3390/foods12091851. 37174389 PMC10177904

[jfds71432-bib-0084] Sagdic, O. , I. Ozturk , H. Cankurt , and F. Tornuk . 2012. “Interaction Between Some Phenolic Compounds and Probiotic Bacterium in Functional Ice Cream Production.” Food and Bioprocess Technology 5, no. 8: 2964–2971. 10.1007/s11947-011-0611-x.

[jfds71432-bib-0085] Saygili, D. , O. Yerlikaya , and A. Akpinar . 2023. “The Effect of Using Different Yeast Species on the Composition of Carbohydrates and Volatile Aroma Compounds in Kefir Drinks.” Food Bioscience 54: 102867. 10.1016/j.fbio.2023.102867.

[jfds71432-bib-0086] Sözeri Atik, D. , B. Gürbüz , E. Bölük , and İ. Palabıyık . 2021. “Development of Vegan Kefir Fortified With Spirulina Platensis.” Food Bioscience 42: 101050. 10.1016/j.fbio.2021.101050.

[jfds71432-bib-0087] Spizzirri, U. G. , M. R. Loizzo , F. Aiello , S. A. Prencipe , and D. Restuccia . 2023. “Non‐Dairy Kefir Beverages: Formulation, Composition, and Main Features.” Journal of Food Composition and Analysis 117: 105130. 10.1016/j.jfca.2023.105130.

[jfds71432-bib-0088] Tanaka, N. 1982. “Toxin Production by *Clostridium botulinum* in Media at pH Lower Than 4.6.” Journal of Food Protection 45, no. 3: 234–237. 30866277 10.4315/0362-028X-45.3.234

[jfds71432-bib-0089] Tang, X. , J. Chen , B. Jiang , Q. Zhu , and R. Zhang . 2023. “Effects of Lactiplantibacillus Plantarum Fermentation on Hydrolysis and Immunoreactivity of Siberian Apricot (*Prunus sibirica* L.) Kernel.” Food Bioscience 53: 102585. 10.1016/j.fbio.2023.102585.

[jfds71432-bib-0090] Taş, N. G. , C. Yılmaz , and V. Gökmen . 2019. “Investigation of Serotonin, Free and Protein‐Bound Tryptophan in Turkish Hazelnut Varieties and Effect of Roasting on Serotonin Content.” Food Research International 120: 865–871. 10.1016/j.foodres.2018.11.051. 31000307

[jfds71432-bib-0091] Teixeira Oliveira, J. , F. Machado da Costa , T. Gonçalvez da Silva , et al. 2023. “Green Tea and Kombucha Characterization: Phenolic Composition, Antioxidant Capacity and Enzymatic Inhibition Potential.” Food Chemistry 408: 135206. 10.1016/j.foodchem.2022.135206. 36528993

[jfds71432-bib-0092] Tireki, S. 2025. “Influence of Water Properties on the Physicochemical and Sensorial Parameters of Water Kefir.” Journal of Culinary Science & Technology 23, no. 3: 579–599. 10.1080/15428052.2024.2303002.

[jfds71432-bib-0093] Torre, L. L. , A. Y. Tamime , and D. D. Muir . 2003. “Rheology and Sensory Profiling of Set‐Type Fermented Milks Made With Different Commercial Probiotic and Yoghurt Starter Cultures.” International Journal of Dairy Technology 56, no. 3: 163–170. 10.1046/j.1471-0307.2003.00098.x.

[jfds71432-bib-0094] Tsai, M. J. , M. C. Cheng , B. Y. Chen , and C. Y. Wang . 2018. “Effect of High‐Pressure Processing on Immunoreactivity, Microbial and Physicochemical Properties of Hazelnut Milk.” International Journal of Food Science & Technology 53, no. 7: 1672–1680. 10.1111/ijfs.13751.

[jfds71432-bib-0095] Tu, C. , F. Azi , J. Huang , X. Xu , G. Xing , and M. Dong . 2019. “Quality and Metagenomic Evaluation of a Novel Functional Beverage Produced From Soy Whey Using Water Kefir Grains.” LWT 113: 108258. 10.1016/j.lwt.2019.108258.

[jfds71432-bib-0096] Uruc, K. , A. Tekin , D. Sahingil , and A. A. Hayaloglu . 2022. “An Alternative Plant‐Based Fermented Milk With Kefir Culture Using Apricot (*Prunus armeniaca* L.) Seed Extract: Changes in Texture, Volatiles and Bioactivity During Storage.” Innovative Food Science & Emerging Technologies 82: 103189. 10.1016/j.ifset.2022.103189.

[jfds71432-bib-0097] Ustaoğlu‐Gençgönül, M. , Ç. Gökırmaklı , B. Üçgül , Y. Karagül‐Yüceer , and Z. B. Guzel‐Seydim . 2024. “Chemical, Microbial, and Volatile Compounds of Water Kefir Beverages Made From Chickpea, Almond, and Rice Extracts.” European Food Research and Technology 250, no. 8: 2233–2244. 10.1007/s00217-024-04533-9.

[jfds71432-bib-0098] Wang, Y. , Y. Xu , Y. Li , et al. 2025. “Flavor Formation in Water Kefir: Metabolic Roles of Microorganisms and Their Regulatory Pathways.” Food Reviews International 41, no. 9: 3552–3568. 10.1080/87559129.2025.2572780.

[jfds71432-bib-0099] Wu, S. , Y. Chen , Z. Chen , et al. 2023. “Antioxidant Properties and Molecular Mechanisms of Lactiplantibacillus Plantarum ZJ316: A Potential Probiotic Resource.” LWT 187: 115269. 10.1016/j.lwt.2023.115269.

[jfds71432-bib-0100] Xu, Y. , F. Zhang , G. Mu , and X. Zhu . 2024. “Effect of Lactic Acid Bacteria Fermentation on Cow Milk Allergenicity and Antigenicity: A Review.” Comprehensive Reviews in Food Science and Food Safety 23, no. 1: e13257. 10.1111/1541-4337.13257. 38284611

[jfds71432-bib-0101] Yanto, T. , K. Karseno , and M. M. Purnamasari . 2015. “The Influence of the Type and Concentration of Sugar on the Physicochemical and Sensory Characteristics of Jelly Drinks.” Jurnal Teknologi Hasil Pertanian 8, no. 2: 123–129.

[jfds71432-bib-0102] Yin, X. , L. Yang , X. Sun , et al. 2024. “Development and Validation of Sensitive and Rapid CRISPR/Cas12‐Based PCR Method to Detect Hazelnut in Unlabeled Products.” Food Chemistry 438: 137952. 10.1016/j.foodchem.2023.137952. 38007952

